# Discovery of an *In Vivo* Chemical Probe for BCL6 Inhibition by Optimization of Tricyclic Quinolinones

**DOI:** 10.1021/acs.jmedchem.3c00155

**Published:** 2023-04-07

**Authors:** Alice C. Harnden, Owen A. Davis, Gary M. Box, Angela Hayes, Louise D. Johnson, Alan T. Henley, Alexis K. de Haven Brandon, Melanie Valenti, Kwai-Ming J. Cheung, Alfie Brennan, Rosemary Huckvale, Olivier A. Pierrat, Rachel Talbot, Michael D. Bright, Hafize Aysin Akpinar, Daniel S. J. Miller, Dalia Tarantino, Sharon Gowan, Selby de Klerk, Peter Craig McAndrew, Yann-Vaï Le Bihan, Mirco Meniconi, Rosemary Burke, Vladimir Kirkin, Rob L. M. van Montfort, Florence I. Raynaud, Olivia W. Rossanese, Benjamin R. Bellenie, Swen Hoelder

**Affiliations:** ^†^Centre for Cancer Drug Discovery and ^‡^Division of Structural Biology, The Institute of Cancer Research, London SM2 5NG, U.K.

## Abstract

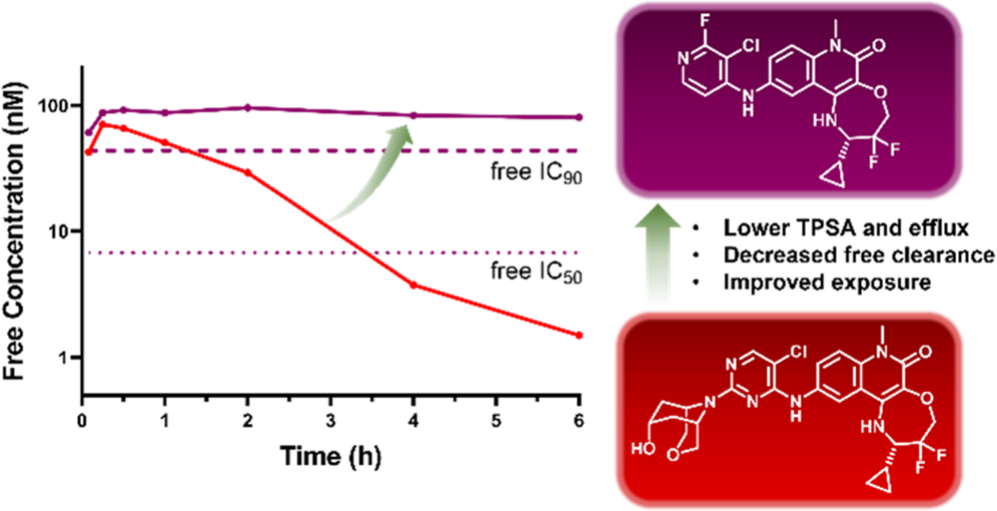

B-cell lymphoma 6 (BCL6) is a transcriptional repressor and oncogenic driver of diffuse large B-cell lymphoma (DLBCL). Here, we report the optimization of our previously reported tricyclic quinolinone series for the inhibition of BCL6. We sought to improve the cellular potency and *in vivo* exposure of the non-degrading isomer, **CCT373567**, of our recently published degrader, **CCT373566**. The major limitation of our inhibitors was their high topological polar surface areas (TPSA), leading to increased efflux ratios. Reducing the molecular weight allowed us to remove polarity and decrease TPSA without considerably reducing solubility. Careful optimization of these properties, as guided by pharmacokinetic studies, led to the discovery of **CCT374705**, a potent inhibitor of BCL6 with a good *in vivo* profile. Modest *in vivo* efficacy was achieved in a lymphoma xenograft mouse model after oral dosing.

## Introduction

The B-cell lymphoma 6 (BCL6) protein is a transcriptional repressor required for the expansion of germinal center B-cells.^1,2^ Expression is highly regulated in normal B-cells allowing for rapid proliferation during somatic hypermutation. Dysregulation of and dependence on BCL6 is regularly observed in B-cell lymphomas.^3,4^ BCL6 acts as a transcriptional repressor to a broad range of genes via the recruitment of corepressors (NCOR, SMRT, or BCOR) to its dimeric BTB domain, which enables binding to key sites on DNA.^5,6^ The protein–protein interaction (PPI) between BCL6 and its corepressors has been targeted as a potential therapy for BCL6-driven lymphomas. Promising small molecule inhibitors of the BTB domain have been reported by us and others with limited suitable *in vivo* candidates for cancer studies.^7−15^

**Figure 1:**
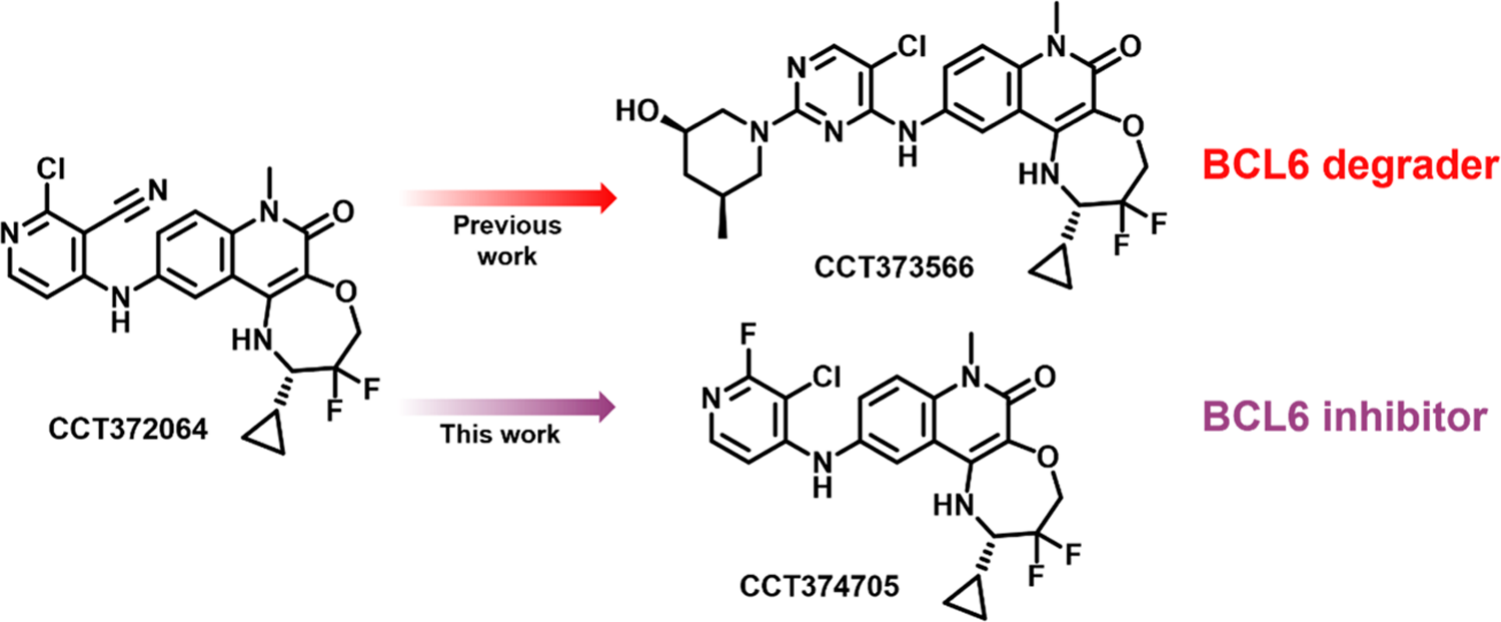
Optimization of **CCT372064**(13) toward BCL6 degradation (**CCT373566**^16^) and inhibition (**CCT374705**).

We recently reported a new tricyclic quinolinone scaffold that demonstrated improved binding affinity to BCL6 (**CCT372064**: BCL6 HTRF IC_50_ = 4.8 nM) compared to our earlier benzimidazolone and quinolinone series (Figure 1).^13^

We then optimized this scaffold for the degradation of BCL6 through modification of the 2-pyrimidine substituent of this core. Degradation required piperidine in this position decorated with either 3-methyl or 4,4-difluoro. Introducing polarity onto piperidine to decrease the log *D* was crucial to lower *in vivo* clearance and also increase the potency of inhibition and degradation. Ultimately, this optimization culminated in the discovery of **CCT373566**, an extremely potent (DC_50_ = 0.7 nM) *in vivo* degrader that demonstrated strong antiproliferative activity in cells but showed only modest *in vivo* efficacy.^16^

In this study, we report the discovery of a potent *in vivo* BCL6 inhibitor. Further optimization of our tricyclic quinolinone series was needed in search of an oral tool compound to validate the role of BCL6 inhibition in tumor models in mice. Initially, we aimed to improve potency through additional exploration of the 2-pyrimidine substituent. However, while potent BCL6 inhibitors (cellular IC_50_ < 20 nM) were discovered, *in vivo* studies were limited by low free compound concentrations. Achieving an optimal pharmacokinetic profile required careful balancing of physicochemical properties that led us to decrease the molecular weight (MW) of our series. The optimization resulted in the discovery of compound **CCT374705**, an inhibitor with potent antiproliferative effects *in vitro* and sustained exposure above the predicted required concentrations.

## Results and Discussion Chemistry

**Scheme 1:**
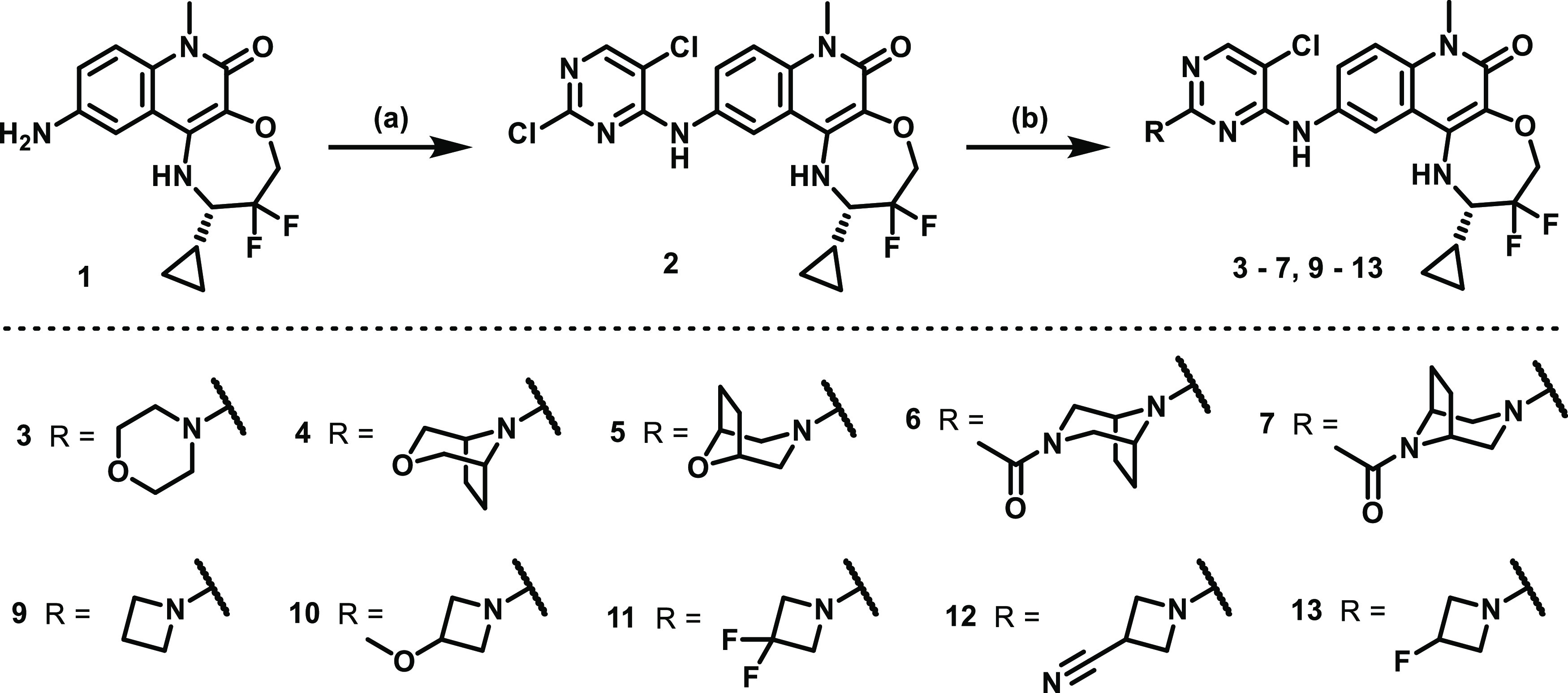
Synthesis of Substituted Pyrimidine Tricyclic Quinolinone Compounds from Tables 2, 4, and 6, Starting from Common Intermediate **1** Reagents and conditions: (a) 2,4,5-trichloropyrimidine or 2,4-dichloro-5-fluoropyrimidine, DIPEA, NMP, 140 °C, 1 h; (b) cyclic amine, DIPEA, NMP or MeCN, 80–140 °C, 1–18 h.

**Scheme 2:**
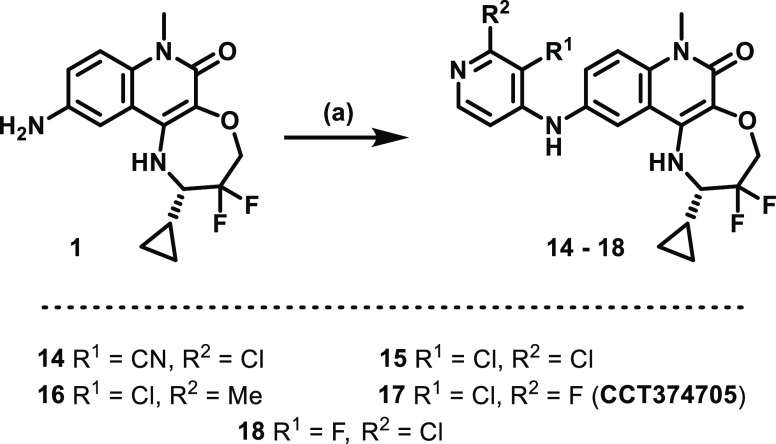
Synthesis of Substituted Pyridine Tricyclic Quinolinone Compounds from Table 8, Starting from Common Intermediate **1** Reagents and conditions: (a) 4-bromo or iodopyridine, Xantphos, Pd_2_(dba)_3_ or Pd(OAc)_2_, cesium carbonate, DMF/toluene, 80 °C, 1–18 h.

**Scheme 3:**
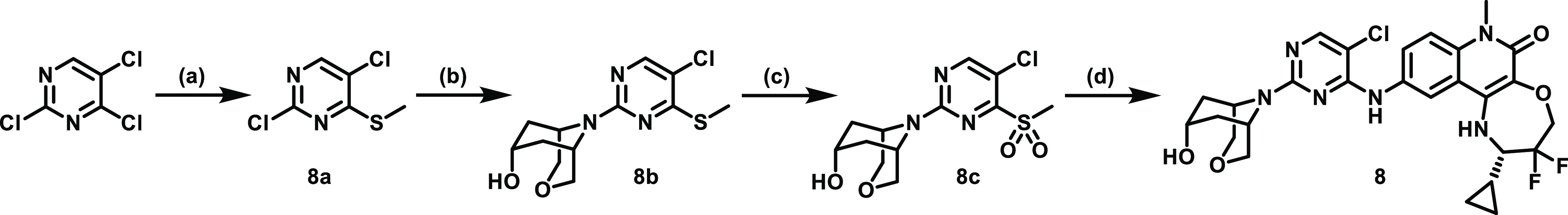
Synthesis of Endo-Hydroxy-Piperidine Containing Compound **8** Reagents and conditions: (a) sodium thiomethoxide, THF/H_2_O, 0 °C to rt, 4 h; (b) endo-7-hydroxy-3-oxa-9-azabicyclo[3.3.1]nonane hydrochloride, DIPEA, isopropanol, 120 °C, 24 h; (c) mCPBA, CH_2_Cl_2_/MeCN, rt, 3 h; (d) **1**, TFA, 2,2,2-trifluoroethanol, 70 °C, 20 h.

**Scheme 4:**
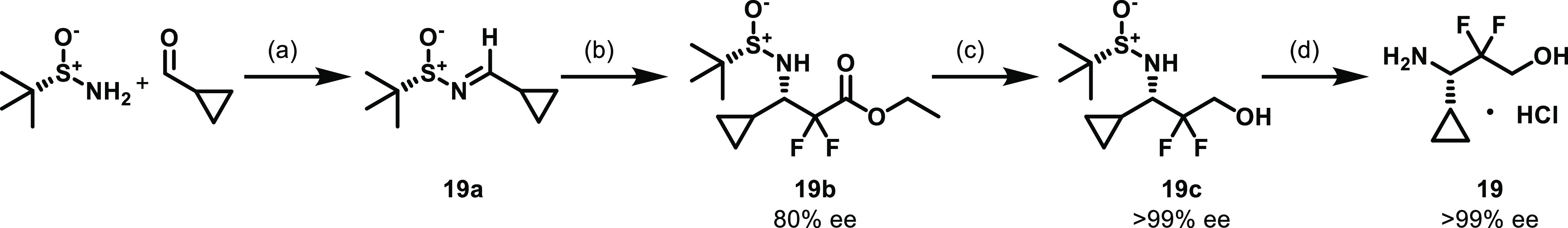
Synthesis of Enantiopure Intermediate 19 Reagents and conditions: (a) MgSO_4_, pyridium *p*-toluenesulfonate, CH_2_Cl_2_, rt, 18 h; (b) Zn, ethyl bromodifluoroacetate, DIBAL-H, THF, 40 °C; (c) NaBH4, MeOH, 0 °C to rt, 1 h; (d) HCl, 1,4-dioxane, rt, 1.5 h.

Final compounds were generally obtained by a single-step nucleophilic aromatic substitution (S_N_Ar) reaction from dichloropyrimidine intermediate **2** (Scheme 1) or, for pyridine substituents, by palladium-catalyzed cross-coupling reactions (Scheme 2). While compound **8** could also be prepared via the sequential S_N_Ar route, yields for the final step were low. As such, a new route was developed (Scheme 3); this required the synthesis of the substituted pyrimidine **8c**, with the final compound prepared under acidic conditions. All compounds were obtained from a common aniline intermediate **1**. The synthesis of **1** has been published;^13^ however, previously, the synthetic route required a chiral SFC separation. Commercially sourced starting material (3S)-3-amino-3-cyclopropyl-2,2-difluoro-propan-1-ol hydrochloride, **19**, was supplied at ∼85% ee. An in-house route to this compound as a single enantiomer was established (Scheme 4).

First, cyclopropanecarboxaldehyde was condensed with Ellman′s (S)-sulfinamide in the presence of magnesium sulfate and catalytic PPTS to yield imine **19a**.^17^ An organozinc reagent was prepared from ethyl bromodifluoroacetate and immediately quenched with **19a** via a Reformatsky reaction to yield **19b**. Initially, this reaction was conducted on a moderate scale (<2 g) using preactivated zinc dust.^18^ To avoid handling excessive quantities of activated zinc dust, an *in situ* method of activation was used on larger scale (>15 g) reactions. In an adapted procedure previously developed for multikilogram scale-up, zinc activation was achieved using DIBAL-H in the presence of a small quantity (5%) of imine.^19^ Slow addition of the remaining imine produced a manageable exotherm during the reaction to **19b**. The Reformatsky reaction showed diastereoselectivity (∼80% ee) similar to the commercially available amino alcohol as seen by ^1^H NMR. Column chromatography could not separate the diastereoisomers at this stage. However, after the reduction of the ester by sodium borohydride to alcohol **19c**, the diastereoisomers were separated by normal phase column chromatography. The *tert*-butylsulfinyl group was removed in acid to yield the >99% ee amino alcohol.

### Improving Potency

To probe the effect of BCL6 inhibition in an *in vivo* xenograft model in mice, we wanted to ensure that we could achieve sustained coverage of our inhibitor. It has been shown previously that to induce an antiproliferative effect in BCL6-high cells, concentrations of degraders or inhibitors of BCL6 must be sustained for several days.^10,13,20^ Based on this *in vitro* antiproliferative data, we hypothesized that it would be necessary to employ a dosing regimen that maintained a free concentration *in vivo* above the free IC_90_, as calculated from our cellular NanoBRET assay, for the duration of our *in vivo* studies. This approach would ensure that we are achieving sufficient and continual target engagement and, therefore, truly testing the therapeutic hypothesis of BCL6 inhibition within a xenograft model.

Our previous studies on chiral piperidine-substituted tricyclic quinolinone analogues led to the discovery of extremely potent degraders of BCL6.^16^ Additionally, during these investigations, we observed striking SAR between piperidine enantiomers. While each pair of piperidine stereoisomers showed binding affinity similar to the BTB domain of BCL6, as measured by our biochemical TR-FRET assay (Table 1), only one isomer was shown to induce degradation of the protein. Optimization led to the discovery of *cis*-3,5-substituted degrader **CCT373566**, which demonstrated subnanomolar cellular degradation of BCL6 and displayed an acceptable pharmacokinetic profile and ADME properties (Table 1). *In vivo* studies of **CCT373566** showed that free plasma concentrations remained above the calculated free DC_50_ levels for over 24 h when dosed at 50 mg/kg. The tricyclic quinolinone substituted with the opposite *cis*-piperidine enantiomer **CCT373567** demonstrated similar biochemical affinity but did not induce degradation of BCL6 and thus appeared as a good candidate BCL6 inhibitor. Unfortunately, while the cellular activity of degrader **CCT373566** was excellent (DC_50_ = 0.7 nM), **CCT373567** showed a large decrease in activity in our NanoBRET cellular inhibition assay (IC_50_ = 25.9 nM). If, as expected, **CCT373567** had *in vivo* pharmacokinetics similar to **CCT373566**, we would require a dose significantly higher than 50 mg/kg to guarantee sustained inhibition of BCL6.

**Table 1:**
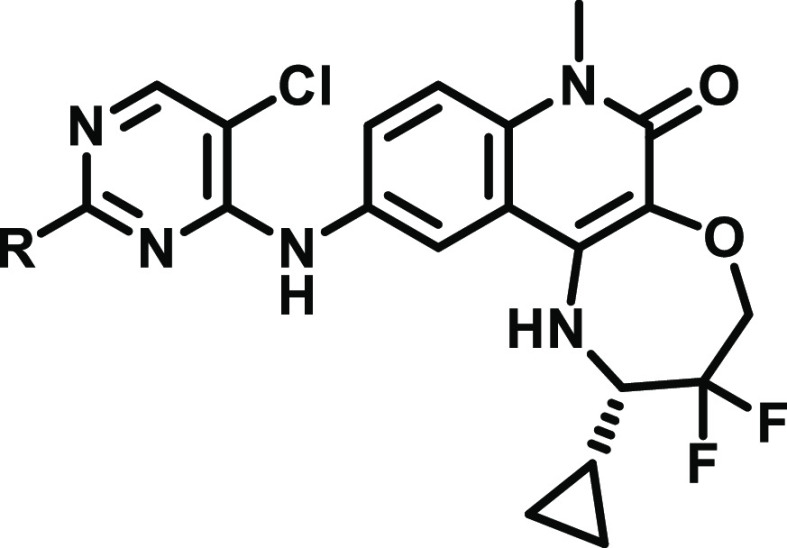

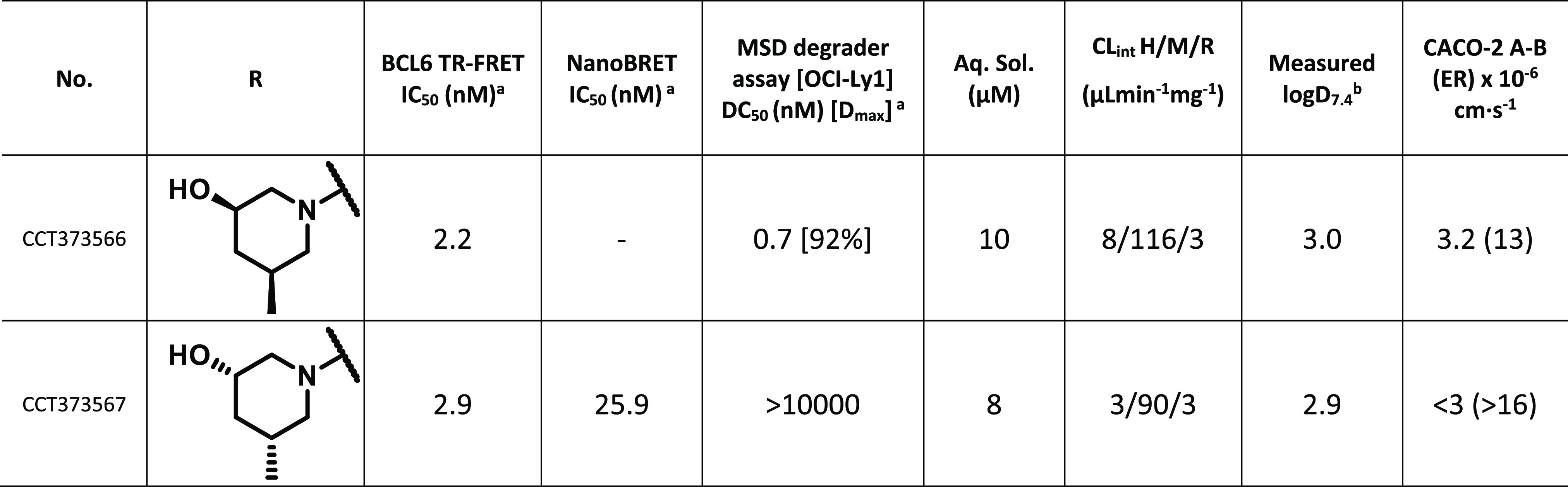
Overview of Degrader **CCT373566** and Inhibitor **CCT373567**(16)

a Data represent the geometric mean of at least three replicates.

b Measured log *D* determined using the Chrom log *D* method.

**Figure 2:**
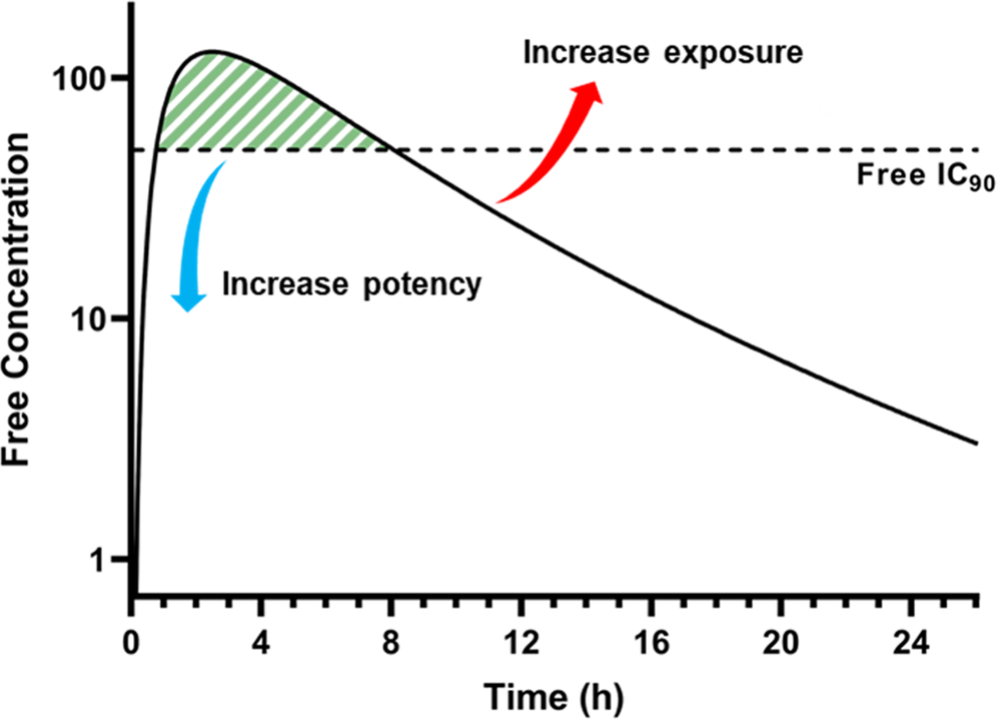
Schematic showing the two methods to increase the amount of time the free concentration of a drug remains above the free IC_90_ (area shaded green).

To improve upon **CCT373567**, we must either improve the cellular potency and/or increase the free exposure of the compound through optimization of PK properties (Figure 2).

Our previous studies on degrader tricyclic quinolinone analogues demonstrated that biochemical potency gains could be achieved via modification of the 2-amino substituent of the pyrimidine ring.^16,20^ Additionally, we showed that modification of this part of the molecule considerably impacts the overall physicochemical and *in vitro* properties.

In our alternate benzimidazolone series, morpholine and piperazine groups were among the most potent inhibitors.^20^ We hypothesized that we could increase the affinity of **CCT373567** through the replacement of the 6-membered piperidine with these other heterocycles.

We tested a morpholine substituent on our tricyclic quinolinone core (**3**, Table 2). This compound showed biochemical affinity similar to BCL6 (3.2 nM) as **CCT373567**, and a modest improvement in mouse microsomal clearance (57 vs 90) was also observed. The morpholine analogue **3** also showed an improvement in permeability and efflux compared to **CCT373567**.

**Table 2:**
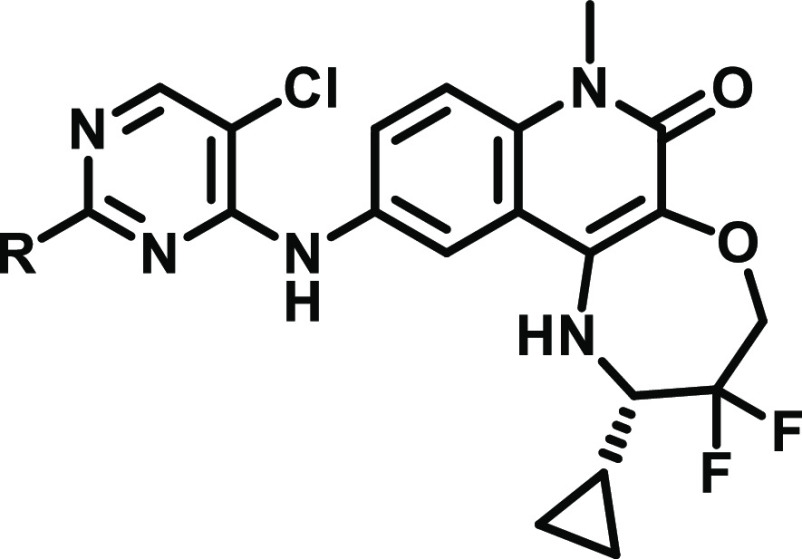

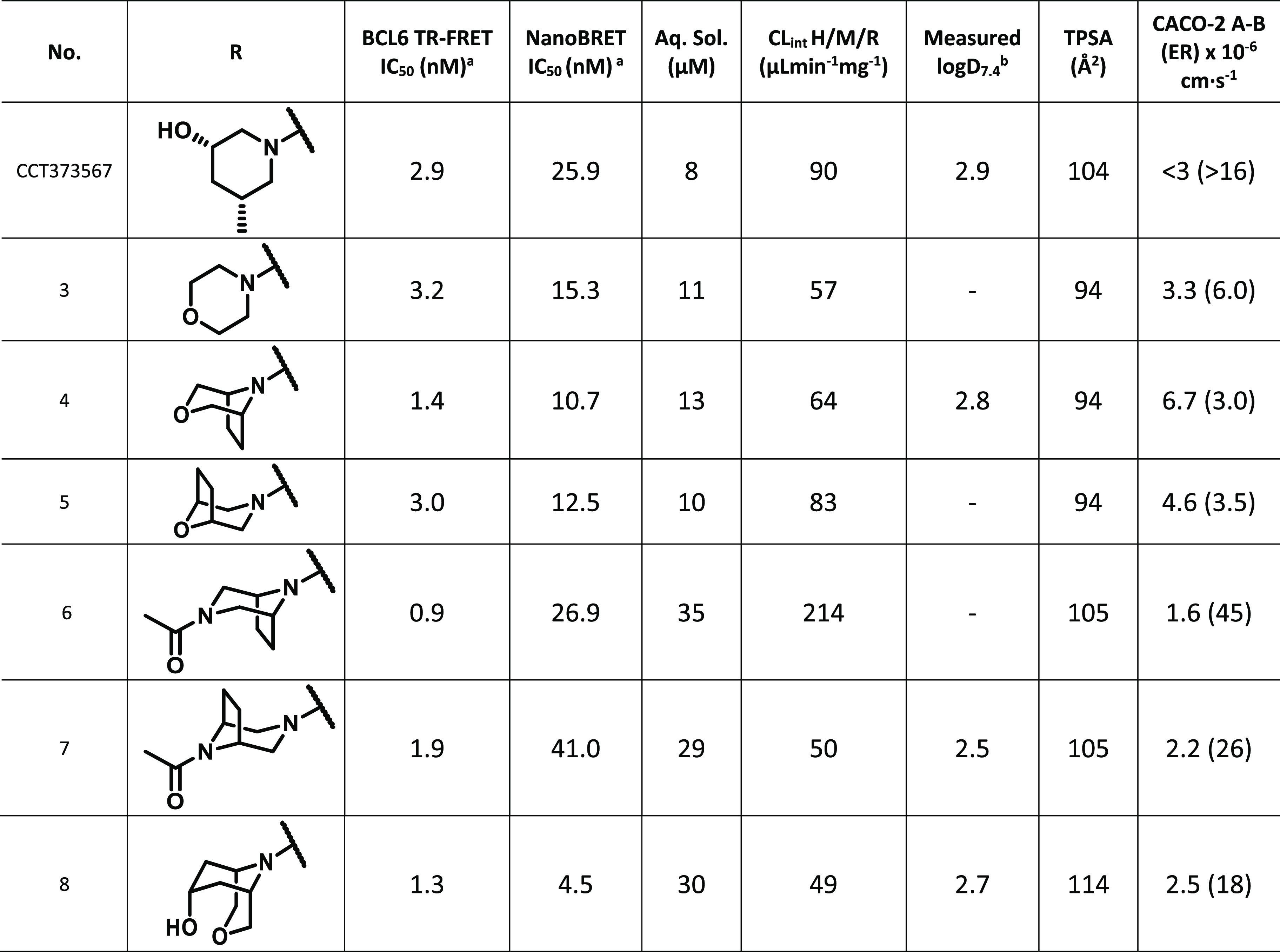
Structure–Activity Relationships of 6-Membered Cyclic Amine-Substituted Pyrimidines

a Data represent the geometric mean of at least three replicates. See Tables S1 and S2 for full statistics.

b Measured log *D* determined using the Chrom log *D* method.

**Figure 3:**
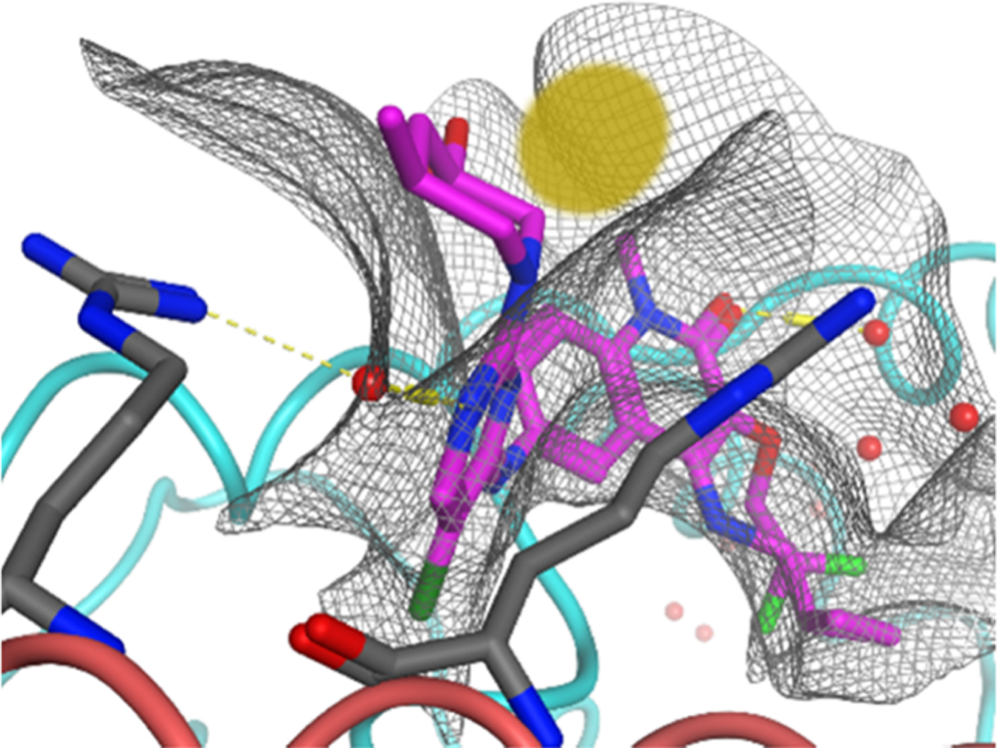
X-ray structure of the BCL6 BTB domain with bound ligand **CCT373566** (PDB: 7QK0, magenta).^16^ The yellow area highlights space in the pocket to be filled with bridged compounds. The backbones of the two BCL6 monomers are colored as blue and pink ribbons. Selected water molecules are shown as red spheres and H-bonds as yellow dashed lines.

By examining the crystal structure of **CCT373566**,^16^ we hypothesized that bridged bicyclic derivatives of morpholines and piperazines could fill the space between the two solvent-exposed surfaces of the binding pocket more effectively and may therefore be more potent toward BCL6 (Figure 3). Additionally, we hoped that increasing the 3-dimensional shape of the molecules could compensate for the loss of solubility that might result from increasing lipophilicity. We explored several bridged morpholine and piperazine analogues (Table 2).

The bridged compounds (**4–7**) were found to be extremely potent in our TR-FRET assay, IC_50_ ≤ 3 nM. However, the transition from biochemical to cellular inhibition varied between morpholine and piperazine compounds. Both isomers of the bridged N-acyl piperazine (**6** and **7**) suffered a large loss in affinity in cells (26.9 and 41.0 nM) and were disappointingly less potent than **CCT373567**. The two bridged morpholine compounds, **4** and **5**, represented our most potent compounds in cells so far, 10.7 and 12.5 nM, respectively. Moreover, the addition of the bridge led to better permeability (6.7/4.6 vs 3.3) and efflux ratio (3.0/3.5 vs 6.0) compared to **3**.

To fill more of the wedge-shaped pocket (Figure 3), we prepared a larger bicycle containing morpholine with an endo-hydroxy-piperidine, i.e., endo-3-oxa-9-azabicyclo[3.3.1]nonan-7-ol (**8**). It was hypothesized that we could maintain good permeability and solubility through the presence of the polar OH group with an intramolecular hydrogen bond. The combination of the larger bridge and H-bond of **8** resulted in a cellular potency of 4.5 nM, with increased solubility and lower mouse microsomal clearance compared to **CCT373567**.

We decided to conduct further profiling *in vivo* on our two most potent cellular compounds to date, **4** and **8**. **8** was our most potent compound in cells and demonstrated good solubility and lower microsomal clearance. Although slightly less potent than **8**, compound **4** exhibited a significantly lower efflux ratio, but the main concern was poorer solubility.

### *In Vivo* Profiling of **4** and **8**

**Figure 4:**
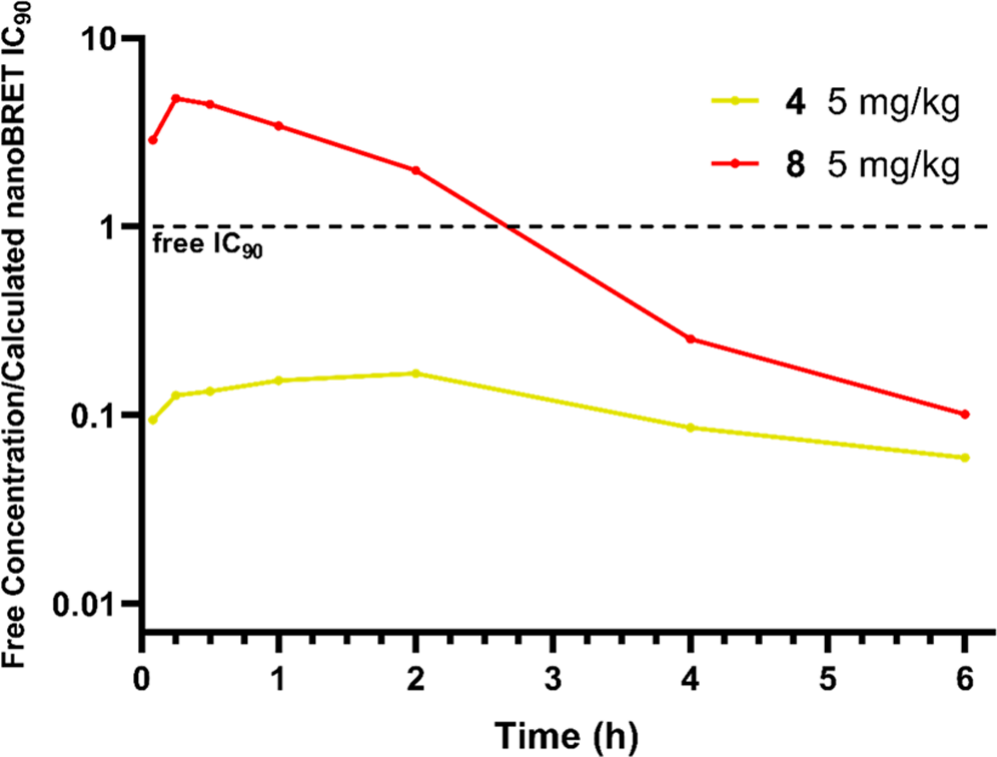
Free mean mouse blood concentrations (nM) divided by the respective calculated free, cellular (from NanoBRET assay) IC_90_ values (nM) of **4** (yellow) and **8** (red) after PO dosing at 5 mg/kg. The dashed, black line represents when a compound is at the free concentration equal to its free IC_90_ value.

We carried out low-dose pharmacokinetic studies, administering at 1 mg/kg i.v. (*n* = 3) and 5 mg/kg p.o. (*n* = 3) in female Balb/C mice. All mice appeared normal post dosing and 24 h post dose. Each compound had a clear solution when formulated, however, **4** came out of the solution as a cloudy suspension just before PO dosing. Consequently, **4** was poorly bioavailable (21%) compared to **8** (66%) (Table 3), resulting in a much lower maximum concentration (*C*_max_). Additionally, **4** had a higher free IC_90_ than **8** and, as a result, the concentration, once corrected for protein binding, never reached this required value when dosed at 5 mg/kg (Figure 4). While both compounds have low total *in vivo* clearance, the lower clearance of **4** compared to **8** (4.9 mL/min/kg vs 7.3 mL/min/kg) leads to a longer half-life (1.12 h vs 0.68 h) (Table 3). However, once corrected for protein binding, the unbound clearance of **4** was higher than **8**, consistent with the results from our *in vitro* microsomal assay. Assuming linear PK, the free concentration of **8** was predicted to remain above the free IC_90_ (as calculated from the NanoBRET assay) for 6 h when orally dosed at 50 mg/kg (Figure S1).

**Table 3:** Pharmacokinetic Properties of Compounds **4** and **8**

no.	BCL6 TR-FRET IC_50_ (nM)^a^	NanoBRET IC_50_ [free IC_50_] (nM)^a^	NanoBRET IC_90_ [free IC_90_] (nM)^a^	AUC_PO_ (6 h) [AUC_PO,u_] (nM·h)	*C*_PO_^max^ [*C*_PO,u_^max^] (nM)	CL [CL_u_] (mL/min/kg)	*t*_1/2_ (h)	*V*_ss_ (L)	*F* (%)	mouse (BALB\c) PPB
**4**	1.4	11 [143]	3.2 [42]	6348 [20]	1567 [5]	4.9 (5% Qh) [1550]	1.12	0.47	21	99.682
**8**	1.3	4.5 [25]	2.7 [15]	13 250 [85]	6904 [45]	7.3 (8% Qh) [1132]	0.68	0.33	66	99.355

a Data represent the geometric mean of at least three replicates. See Tables S1 and S2 for full statistics.

Despite the maximum free concentration (unbound *C*_max_) of **8** reaching well above the free IC_90_, the short half-life (<1 h) results in lower concentrations of the compound at later time points. Conversely, the unbound *C*_max_ of **4** is low and limited by poor solubility and bioavailability. The poorer free cellular potency of **4** also results in the requirement for higher concentrations of compound needed to exceed the free IC_90_. Neither compound fulfilled our desired pharmacokinetic profile. We hypothesized that we needed to target a compound with a profile similar to **8** but with decreased efflux. Alternatively, a compound with properties similar to **4** but with increased potency and solubility could also fulfill our desired target profile. Both compounds also show high mouse microsomal clearance, which could also be improved, although total *in vivo* clearance is low.

### Reducing Molecular Weight to Decrease Efflux

The PK results of **4** and **8** demonstrated that the properties of our tricyclic inhibitors needed to be carefully balanced to improve coverage above the free IC_90_.

Of the compounds tested in Table 2, **8** is the most potent in cells and has the lowest microsomal clearance and comparatively good solubility. The major disadvantage compared to the other compounds tested is low permeability and high efflux. Thus, to improve upon **8**, we should aim to maintain potent cellular IC_90_ and good solubility while optimizing for a decreased efflux. The compounds in Table 2 demonstrate that while log *D* has some influence on the efflux, the best indicator of poor permeability and high efflux in this series is an increased topological polar surface area (TPSA). Therefore, our next aim was to reduce the TPSA as much as possible to reduce the efflux while also ensuring that compounds remain soluble enough for dosing at higher concentrations. We hypothesized that lowering the molecular weight (MW) of our amine-based pyrimidine substituents would be a good strategy to allow us to decrease the TPSA without decreasing solubility. Reducing the MW of a compound reduces the need for the inclusion of polar solvating groups to compensate for additional lipophilic groups (e.g., methylene groups of morpholine and piperazine). Azetidine represents the lowest MW of possible cyclic amine substituents, and we investigated a number of examples (Table 4).

**Table 4:**
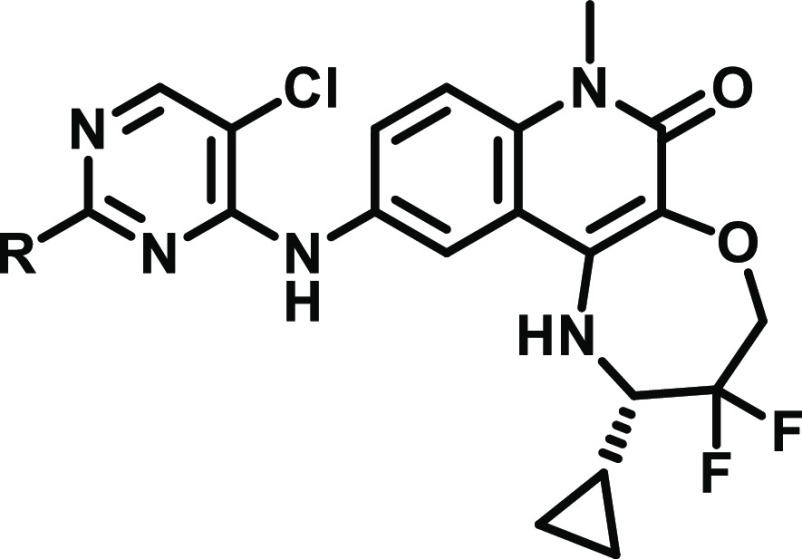

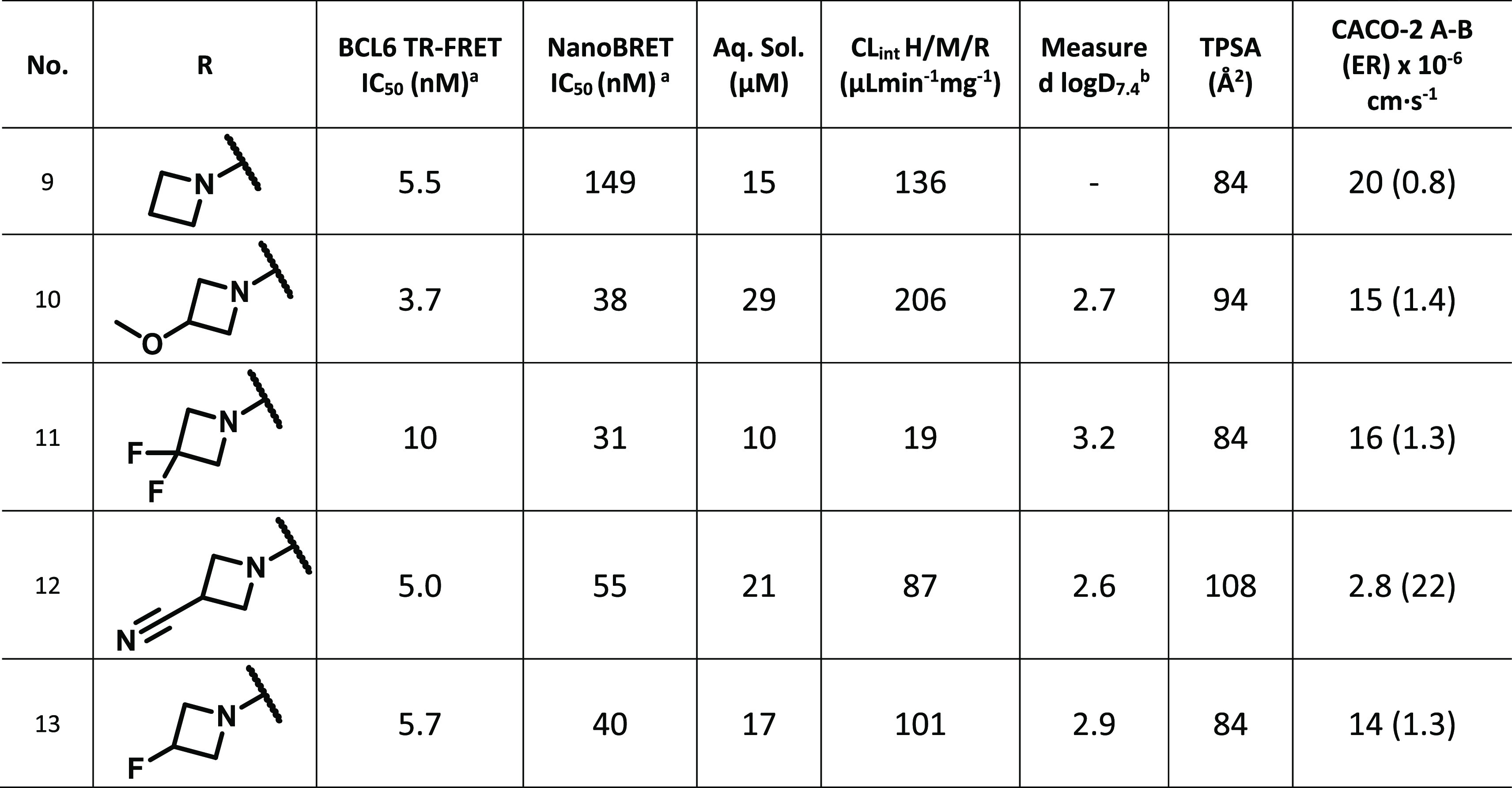
Structure–Activity Relationships of 4-Membered Cyclic Amine-Substituted Pyrimidines

a Data represent the geometric mean of at least three replicates. See Tables S1 and S2 for full statistics.

b Measured log *D* determined using the Chrom log *D* method.

The azetidines remained very potent in our biochemical TR-FRET assay (≤10 nM), however, there was a small general decrease in cellular potency compared to the six-membered cyclic amines of Table 2. Pleasingly, as hypothesized, the caco-2 data was found to correlate better with TPSA than log *D*, suggesting that our strategy of decreasing TPSA does lead to improvement in permeability and efflux. An example of this is the comparison of 3-methoxy-substituted azetidine, **10**, with 3-cyano azetidine, **12**. Both were measured to have a similar log *D*, 2.7 and 2.6, although there was a larger difference in TPSA, 94 and 108, respectively. Whereas **10** showed good permeability (15 × 10^–6^ cm·s^–1^) and a low efflux ratio (1.4), **12** was poorly permeable (2.8 × 10^-6^ cm·s^–1^) and had a substantially higher efflux ratio (22).

Unfortunately, the microsomal clearance in the mouse of the azetidine series was generally poorer than the 6-membered rings, except for the 3,3-difluoroazetidine, **11**, which had the lowest microsomal clearance measured to date. The difluoro analogue was also the most potent azetidine in our cellular NanoBRET assay (31 nM), with a low efflux ratio. We therefore chose **11** as the best representative of this subseries to test *in vivo*.

### *In Vivo* Profiling of **11**

**Figure 5:**
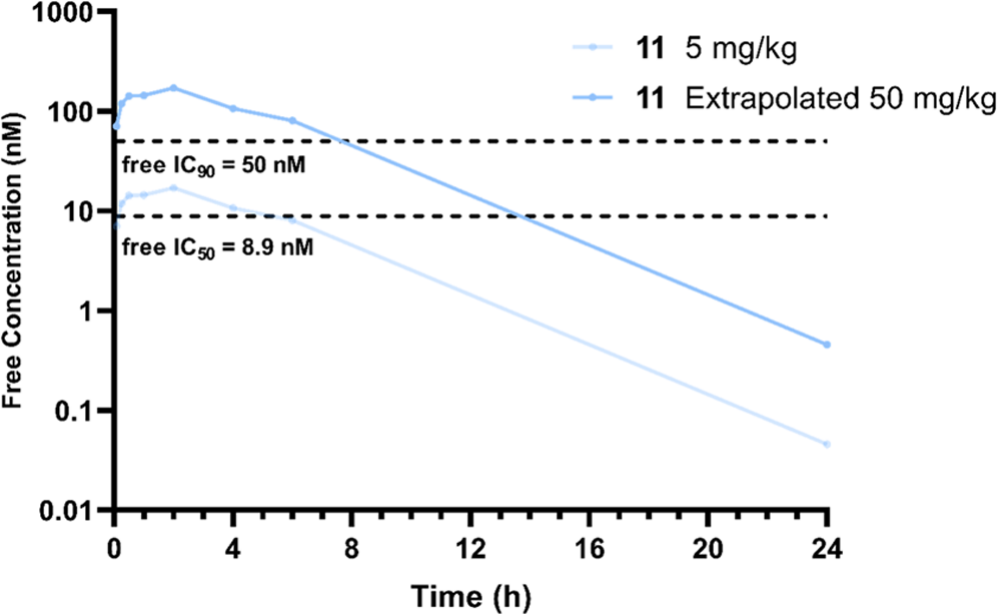
Free mean mouse blood concentrations (nM) of **11** after PO dosing at 5 mg/kg and extrapolated (assuming linear PK) to 50 mg/kg PO dosing. The dashed, black lines represent the calculated free, cellular (from NanoBRET assay) IC_50_ and IC_90_ values (nM).

A pharmacokinetic study, dosing at 1 mg/kg i.v. (*n* = 3) and 5 mg/kg p.o. (*n* = 3), was carried out in female Balb/C mice. All mice appeared normal post dosing and 24 h post dose. The half-life of **11** was increased compared to **8**, despite a lower volume of distribution, due to a drastic improvement in total *in vivo* clearance (Table 5). As observed previously for **4** and **8**, there was a good correlation between our total microsomal clearance measured *in vitro* and our unbound clearance *in vivo*. However, while the low solubility of **11** did not result in precipitation in the dosing solution, the bioavailability was moderate. This, alongside an increase in plasma protein binding, leads to a reduced free concentration, *C*_max_, compared to the more potent inhibitor **8**. Despite this, the decreased total and free clearance led to the highest free drug concentrations at longer time periods to date (Figure S2). Importantly, the free concentration of **11** was expected to remain above the free NanoBRET IC_90_ for ∼8 h when dosed at 50 mg/kg, assuming linear PK, representing an improvement compared to all previous *in vivo* experiments (Figure 5).

**Table 5:** Pharmacokinetic Properties of Compound **11**

no.	BCL6 TR-FRET IC_50_ (nM)^a^	NanoBRET IC_50_ [free IC_50_] (nM)^a^	NanoBRET IC_90_ [free IC_90_] (nM)^a^	AUC_PO_ (6 h) [AUC_PO,u_] (nM·h)	*C*_PO_^max^ [*C*_PO,u_^max^] (nM)	CL [CL_u_] (mL/min/kg)	*t*_1/2_ (h)	*V*_ss_ (L)	*F* (%)	mouse (BALB\c) PPB
**11**	10	31 [8.9]	175 [50]	36 041 [42]	8821 [10]	0.9 (1% Qh) [776]	4.3	0.22	39	99.884

a Data represent the geometric mean of at least three replicates. See Tables S1 and S2 for full statistics.

### Removing Substituents to Increase Solubility

Despite the improvement in coverage gained by **11**, we aimed to find a compound that could give us an even longer time above IC_90_*in vivo*. The initial free concentration of the difluoroazetidine-containing compound was limited by bioavailability, and we thus sought to improve the solubility further without drastically modifying the TPSA or log *D*. We hypothesized that we could achieve this by exploring compounds with even lower MW. Interestingly, our previously reported inhibitor **CCT372064** (**14**) (Figure 1) contained no substituent in the 2-position of pyridine, has a considerably lower MW than **11**, and yet was found to be reasonably potent in both our biochemical and cellular assays (Table 6).^13^ Despite this, no *in vivo* experiments were conducted due to poor permeability and high efflux. As the potency of **CCT372064** was achieved with 6-chloro and 5-nitrile substitutions of pyridine, we investigated other substituted pyridines (Table 6).

**Table 6:**
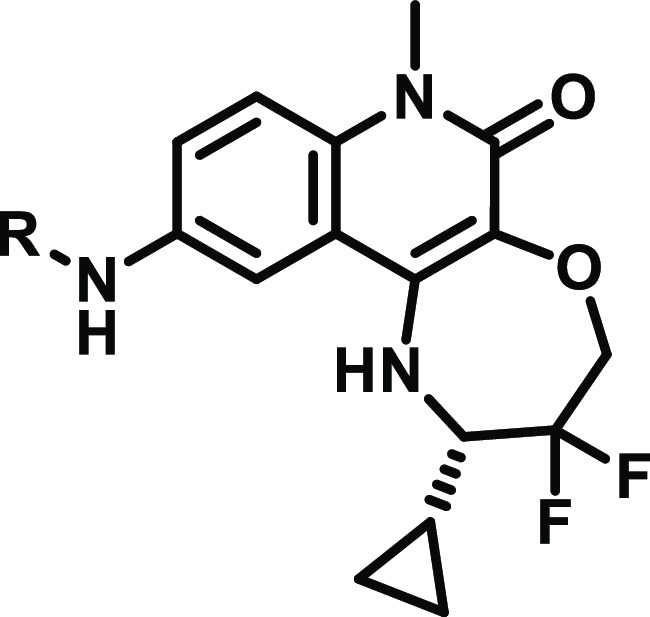

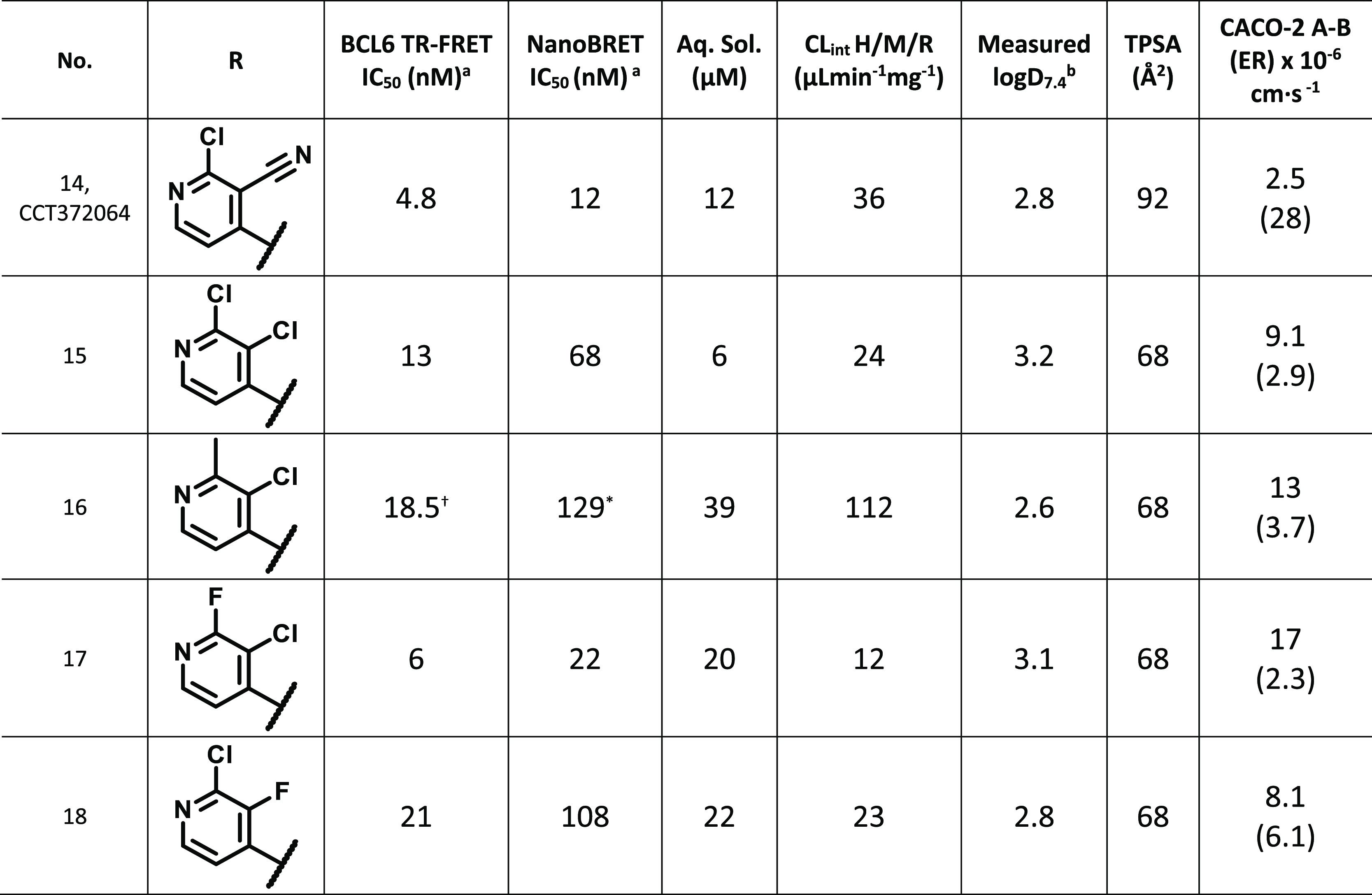
Structure–Activity Relationships of 5- and 6-Substituted Pyridines^c^

a Data represent the geometric mean of at least three replicates. See Tables S1 and S2 for full statistics.

b Measured log *D* determined using the Chrom log *D* method.

c ^†^ indicates *n* = 1 and * indicates *n* = 2.

We sought to lower the TPSA of **CCT372064** by replacing 5-nitrile with a 5-Cl substituent, **15**. Pleasingly, this change did reduce the TPSA (68 vs 92), and a large improvement in permeability (9.1) and efflux (2.9) was observed for **15**. Unfortunately, despite the improved permeability of the dichloro compound, there was a 5-fold decrease in the cellular potency compared to **CCT372064**. Additionally, the solubility was poor (6 μM).

We decided to investigate the 6-position with a larger (methyl – **16**) and smaller (fluoro – **CCT374705** (**17**)) substitution. The addition of a 6-methyl group to our inhibitor **16** did not have the desired outcome, the cellular potency decreased (129 nM), and, in addition, the microsomal clearance was significantly increased (112). The smaller fluoro substitution of **CCT374705** conferred similar biochemical and cellular potencies (6 and 22 nM) to **CCT372064**. The 5-chloro-6-fluoro substituted compound showed good permeability (17) and a low efflux ratio (2.3). We then decided to swap the two substituents to see if we could find any improvement. The 6-chloro-5-fluoro substituted pyridine, **18**, was less potent and had poorer permeability and efflux compared to **CCT374705**.

Therefore, the best combination of 5,6-substitution was found to be 5-chloro-6-fluoropyridine **CCT374705**, demonstrating good potency in our biochemical (6 nM) and cellular (22 nM) assays. In addition to the good potency of **CCT374705**, the 5-chloro-6-fluoro substitution displayed the lowest microsomal clearance of all our inhibitors to date (12).

**Figure 6:**
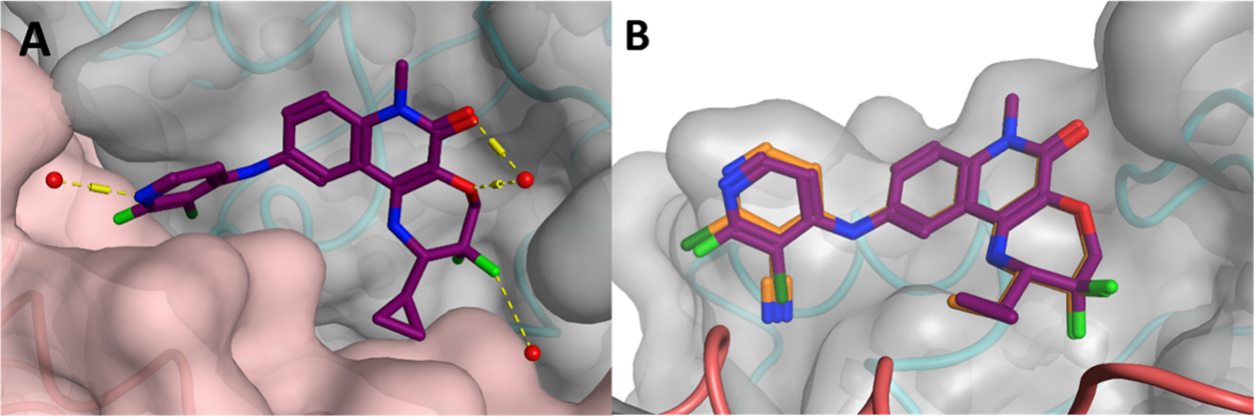
(A) X-ray structure of the BCL6 BTB domain with bound ligand **CCT374705** (PDB: 8C78, purple). (B) Overlaid X-ray structure of the BCL6 BTB domain with bound ligands **CCT372064** (PDB: 7Q7R, orange) and **CCT374705** (PDB: 8C78, purple), the pyridines of both compounds are found to reside in the same position. In both panels, the individual surfaces of the two BCL6 monomers are shown as a gray or pink transparent surface, with the backbones colored as blue and pink ribbons. Selected water molecules are shown as red spheres and H-bonds as yellow dashed lines.

The crystal structure of **CCT374705** bound to BCL6 was solved and showed an identical binding conformation as **CCT372064** (Figure 6). The key interactions, as seen in all previously reported tricyclic quinolone structures, were maintained (pyridine sandwiched between Tyr58 and Asn21, with a π-π interaction with Tyr58, H-bond interactions with Met51, Ala52, and Glu115, and the 7-membered ring filling a subpocket defined by residues His14, Asp17, Val18, and Cys53 of BCL6) (Figure S3). The two pyridines of **CCT374705** and **CCT372064** are well aligned, with the 5,6-substitutions (Cl, F and CN, Cl) occupying the same space in both structures, pointing toward Leu25.

Due to the combination of good cellular potency and promising *in vitro* PK properties, we chose to conduct a pharmacokinetic study on **CCT374705** to see if these favorable properties translated *in vivo*. Safety profiling of **CCT374705** was carried out, and all targets (78) showed a *K*_d_ above 1 μM, although some targets (11/78) did show activity at concentrations below 10 μM (see Supporting Information). A kinase panel (468) confirmed selective activity and minimal off-target interactions.

### *In Vivo* Profiling of **CCT374705**

**Figure 7:**
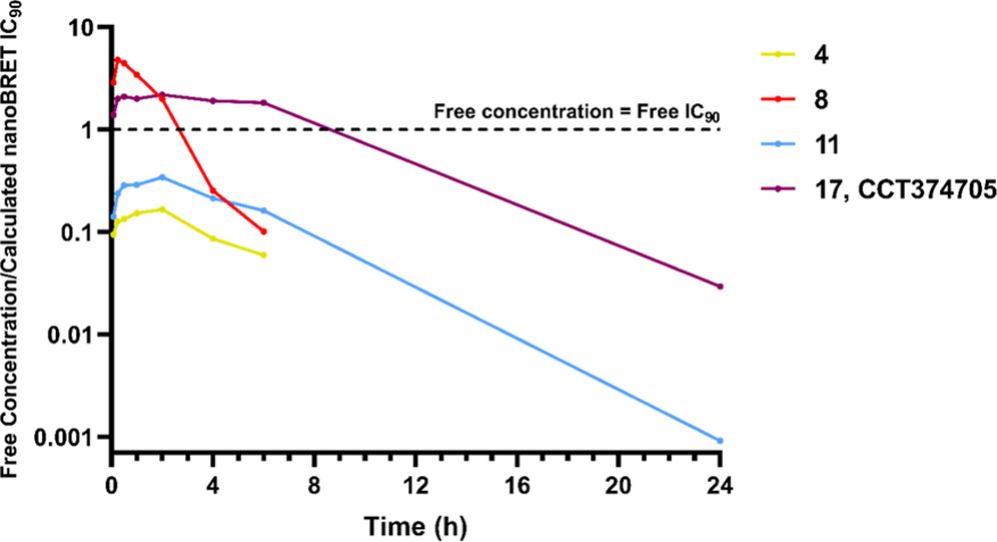
Free mean mouse blood concentrations (nM) divided by the respective calculated free, cellular (from NanoBRET assay) IC_90_ values (nM) of **8** (red), **4** (yellow), **11** (blue), and **CCT374705** (**17**) (purple) after PO dosing at 5 mg/kg. The dashed, black line represents when a compound is at the free concentration equal to its free IC_90_ value.

A pharmacokinetic study, dosing at 1 mg/kg i.v. (*n* = 3) and 5 mg/kg p.o. (*n* = 3), was carried out in female Balb/C mice. All mice appeared normal post dosing and 24 h post dose. The oral bioavailability of **CCT374705** was moderate (48%), only showing a modest increase on that of **12**. However, this improvement, combined with low total clearance and the lowest plasma protein binding (ppb) measured thus far, resulted in the highest free concentrations *in vivo* (Figure S1). The unbound clearance was improved compared to all previously tested compounds leading to the best half-life and longest exposure above the free IC_90_ observed (Table 7). The free concentration was found to remain above the calculated free NanoBRET IC_90_ for over 8 h when orally dosed at 5 mg/kg (Figure 7).

**Table 7:** Pharmacokinetic Properties of All Compounds Tested

no.	BCL6 TR-FRET IC_50_ (nM)^a^	NanoBRET IC_50_ [free IC_50_] (nM)^a^	NanoBRET IC_90_ [free IC_90_] (nM)^a^	AUC_PO_ (6 h) [AUC_PO,u_] (nM·h)	*C*_PO_^max^ [*C*_PO,u_^max^] (nM)	CL [CL_u_] (mL/min/kg)	*t*_1/2_ (h)	*V*_ss_ (L)	F (%)	mouse (BALB\c) PPB
**4**	1.4	11 [3.2]	143 [42]	6348 [20]	1567 [5]	4.9 (5% Qh) [1550]	1.12	0.47	21	99.682
**8**	1.3	4.5 [2.7]	25 [15]	13 250 [85]	6904 [45]	7.3 (8% Qh) [1132]	0.67	0.33	66	99.355
**11**	10	31 [8.9]	175 [50]	36 041 [42]	8821 [10]	0.9 (1% Qh) [776]	2.69	0.22	39	99.884
**17, CCT374705**	6.0	22 [6.8]	140 [44]	42 883 [307]	8652 [62]	1.6 (2% Qh) [223]	2.94	0.39	48	99.283

a Data represent the geometric mean of at least three replicates. See Tables S1 and S2 for full statistics.

The drastic increase in free concentration at later time points was a significant improvement on all previous *in vivo* results. The antiproliferative activity of **CCT374705** was tested in 14-day assays in a range of BCL6-dependent (HT, Karpas 422, SU-DHL-4, and OCI-Ly1) and independent (OCI-Ly3) cell lines (Table 8). The most potent effect was seen in both OCI-Ly1 and Karpas 422 cell lines, where sub-100 nM GI_50_ was observed. As seen with our previously reported inhibitors, despite being high BCL6-expressing, the cell lines HT and SU-DHL-4 were less sensitive to inhibition of BCL6.^13,16^

**Table 8:** Antiproliferative Activity of CCT374705

no.	BCL6 TR-FRET IC_50_ (nM)	NanoBRET IC_50_ (nM)	OCI-Ly1 GI_50_ (nM)	Karpas 422 GI_50_ (nM)	HT GI_50_ (nM)	SU-DHL-4 GI_50_ (nM)	OCI-Ly3 GI_50_ (nM)
**17**, **CCT374705**	6.0	22	38.5	14.9	545	1380	1850

**Figure 8:**
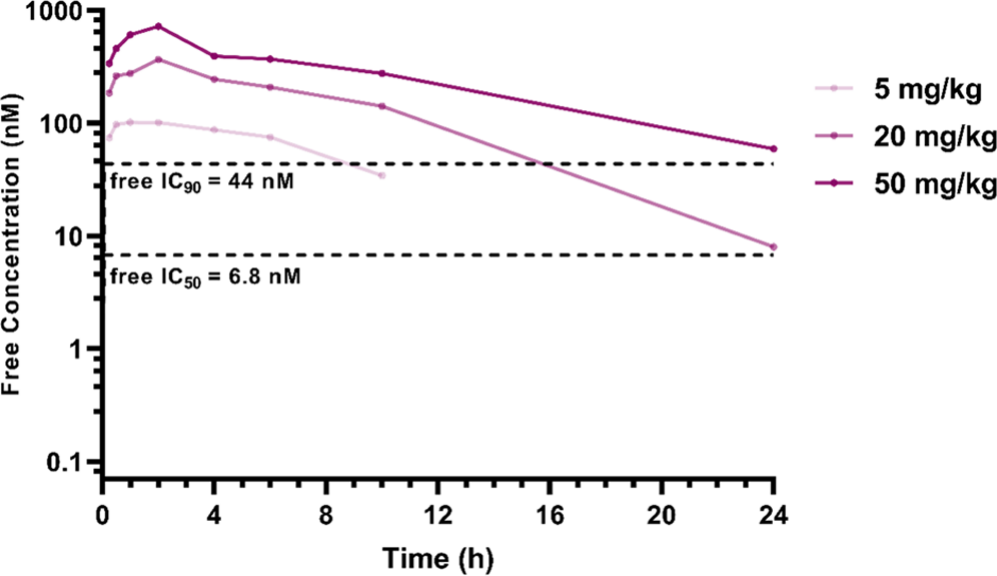
Free mean mouse blood concentrations (nM) of **CCT374705** after PO dosing at 5 mg/kg (lightest purple), 20 and 50 mg/kg (darkest purple). The dashed, black lines represent the calculated free, cellular (from NanoBRET assay) IC_50_ and IC_90_ values (nM).

We performed a PK linearity study in SCID mice with **CCT374705**, dosed at 5 (*n* = 3), 20 (*n* = 3), and 50 (*n* = 3) mg/kg p.o. in a solution formulation previously developed for degrader **CCT373566** (Figure 8).^16^ A linear increase in exposure was observed with increasing dose, and we found that free concentrations of **CCT374705** above the free IC_90_ were achieved at 20 and 50 mg/kg dosing for over 15 and 24 h, respectively.

**Figure 9:**
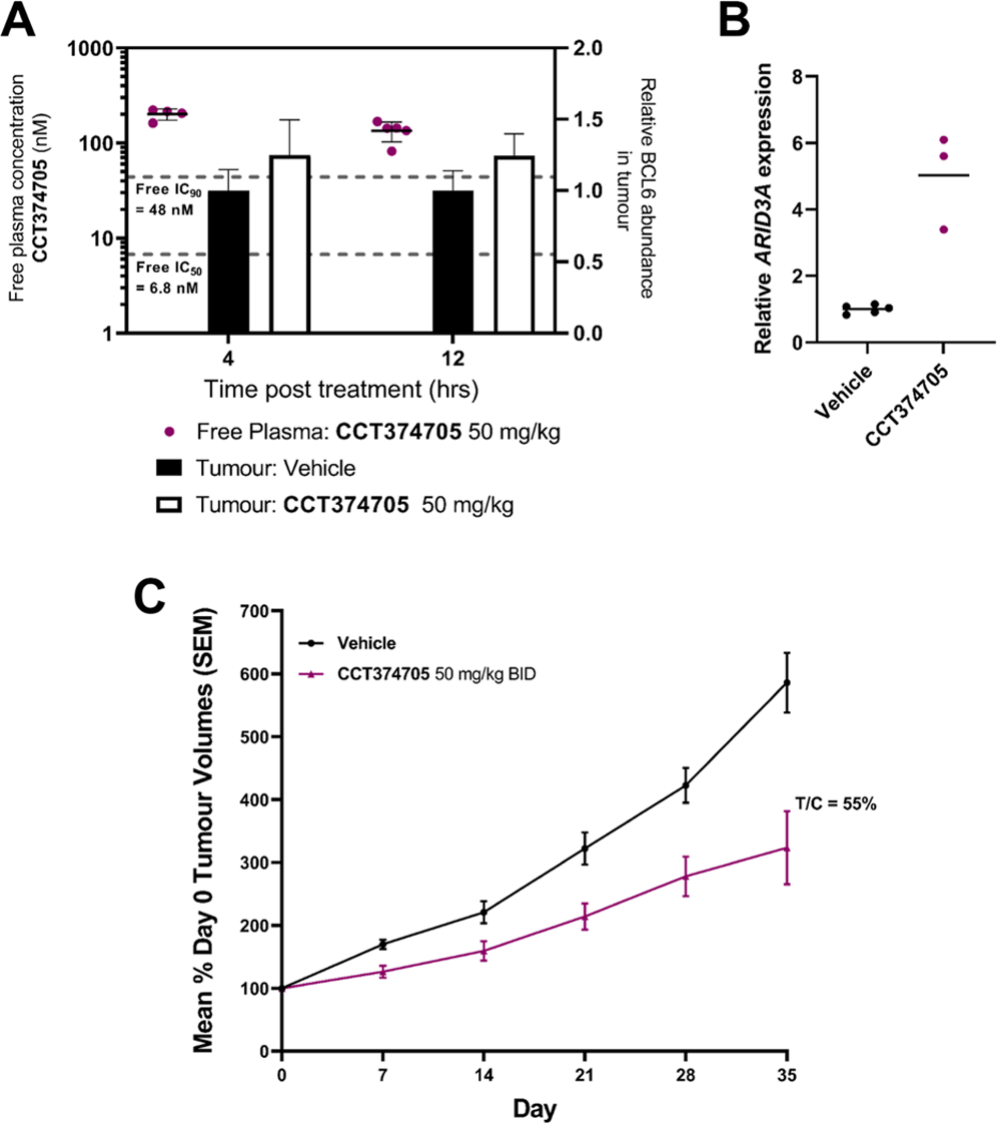
*In vivo* efficacy of **CCT374705**. (A) The PK/PD study with **CCT374705** at 50 mg/kg po. BCL6 levels in the tumor were quantified using capillary electrophoresis and normalized to a GAPDH loading control and are shown as black (vehicle-treated) or white (compound-treated) bars. Free compound levels at 4 and 12 h are shown (purple dots); the dashed, black lines represent the calculated free, cellular (from NanoBRET assay) IC_50_ and IC_90_ values (nM). (B) Relative ARID3A mRNA levels after treatment with **CCT374705** (purple) at 50 mg/kg PO as measured by TaqMan PCR assay. Sampling took place at 12 h post dose. (C) Tumor growth during the efficacy study with **CCT374705** at 50 mg/kg PO BID for 35 days. Tumor xenografts were prepared by subcutaneous injection of 1 × 10^7^ Karpas 422B cells in female SCID mice, with dosing of compound commencing 21 days after injection, to mice with xenografts of ∼0.5 cm^3^, as described in more detail in the Supporting Information. Sampling took place at 4 and 12 h post dose. All experiments were carried out according to the U.K. guidelines for animal experimentation.

The *in vitro* results established that **CCT374705** showed the strongest antiproliferative effect in Karpas 422 cells so an *in vivo* study was undertaken to determine if the promising *in vitro* efficacy could be replicated in a Karpas 422 xenograft model in mice. The compound was dosed orally (50 mg/kg, bid, *n* = 10) for 35 days and was well tolerated with no body weight losses observed. Post treatment analysis showed that free concentrations of **CCT374705** remained well above calculated free IC_90_ for over 12 h post dose (Figure 9A). Unlike in the case of degraders (depletion of BCL6), there is no obvious biomarker for inhibitors of BCL6. We used quantitative RT-PCR to measure gene expression changes in a panel of known BCL6 target genes in different DLBCL lines (data not shown) and found that ARID3A was consistently derepressed. We were able to detect a significant increase in ARID3A mRNA expression in our CCT374705-treated Karpas 422 tumor xenografts, indicating that we were achieving modulation of BCL6 activity (Figure 9B).

Despite sustained exposure of **CCT374705** and clear *in vivo* target engagement, as demonstrated by ARID3A mRNA levels, only a modest slowing of tumor growth was observed compared to the vehicle control group (Figure 9C). After 35 days, the tumor growth inhibition ratio (T/C) of the **CCT374705-**treated group was 0.55.

## Conclusions

The aim of this work was to find a potent compound to investigate the effect of BCL6 inhibition in an *in vivo* mouse model. Our primary aim was to optimize the cellular potency of our previously published inhibitor **CCT373567**, the non-degrading isomer of our potent BCL6 degrader **CCT373566**. While we were able to improve the cellular potency of our inhibitors through the addition of polarity on our 2-pyrimidine substituents, we struggled to balance the increase in polarity with pharmacokinetic performance, particularly efflux (**8**). We decided to focus on the reduction of TPSA, which was found to be the property best correlated to our *in vitro* efflux ratios. Due to the MW of our compounds, the removal of polarity negatively affected our physicochemical properties and solubility, particularly preventing adequate dosing (**4**). We decided to reduce the MW of our inhibitors, switching from 6-membered piperidines and morpholines to 4-membered azetidines. Pleasingly, where TPSA was decreased, the azetidine-substituted compounds did show a decrease in efflux. Despite a decrease in potency, **11** showed improved exposure compared to **8** at later time points due to lower *in vivo* clearance. However, total and free concentrations were limited by low bioavailability, and we decided to further decrease the MW of our inhibitors to ensure higher solubility. We focused on pyridine-containing compounds substituted in both the 5- and 6-positions, analogous to our previously reported tricyclic quinolinone inhibitor **CCT372064**. The Cl- and F-substituted pyridine-containing compound **CCT374705** had the best balance of properties, including increased solubility and microsomal clearance similar to **CCT374284**. The free concentrations *in vivo* were the best seen for all our inhibitors, with the free concentration of **CCT374705** remaining above the free IC_90_ for over 24 h when dosed at 50 mg/kg. Notably, it demonstrated significantly improved unbound *in vivo* clearance compared to all previously tested compounds. Ultimately, we found that optimization of the pharmacokinetic profile of our inhibitors through decreases in molecular weight led to better *in vivo* inhibitors, regardless of any reduction in potency.

We chose to progress **CCT374705** to an efficacy study using a Karpas 422 xenograft model, the cell line in which **CCT374705** had the most antiproliferative effect. The goal of this work was to study the effect of BCL6 inhibition *in vivo*. To assess this, we wanted to ensure complete coverage of our inhibitor (>IC_90_) for the entirety of the study. To achieve this high level of sustained exposure, the study was conducted with a twice-daily 50 mg/kg oral dosing regimen. We were able to confirm target engagement with BCL6 by measuring an increase in ARID3A mRNA expression. PK/PD analysis showed that free concentrations of **CCT374705** remained well above the free IC_90_ for over 12 h post dose. However, similarly to our previously reported efficacy results with our BCL6 degrader **CCT373566**, only moderate *in vivo* efficacy was observed.

Having optimized the *in vivo* pharmacokinetic profile of **CCT374705**, this compound is a suitable probe to examine the role and function of BCL6 in diseases within mice models.

## Experimental Section

All *in vivo* experiments were carried out according to the UK guidelines for animal experimentation. Cell lines were supplied by Public Health England, UK (ECACC). Cell lines were authenticated by STR profiling and were routinely screened for *Mycoplasma* using an in-house PCR-based assay.

### General Synthetic Information

All anhydrous solvents and reagents were obtained from commercial suppliers and used without further purification. Evaporation of solvent was carried out using a rotary evaporator under reduced pressure at a bath temperature of up to 60 °C. Flash column chromatography was carried out using a Biotage purification system using SNAP KP-Sil or Sfar cartridges or on reverse-phase mode using SNAP Ultra C18 cartridges. Semipreparative separations were carried out using a 1200 Series Preparative HPLC over a 15-min gradient elution. Microwave-assisted reactions were carried out using a Biotage Initiator microwave system. The final compounds were purified to ≥95% purity. NMR data was collected on a Bruker Avance 500 spectrometer equipped with a 5 mm BBO/QNP probe or on a Bruker Avance Neo 600 spectrometer equipped with a 5 mm TCI Cryo-Probe. NMR data is presented in the form of chemical shift δ (multiplicity, coupling constants, integration) for major diagnostic protons, given in parts per million (ppm) relative to tetramethylsilane (TMS), referenced to the internal deuterated solvent. HRMS was assessed using an Agilent 1200 series HPLC and a diode array detector coupled to a 6120 time-of-flight mass spectrometer with a dual multimode APCI/ESI source or on a Waters Acquity UHPLC and a diode array detector coupled to a Waters G2 QToF mass spectrometer fitted with a multimode ESI/APCI source.

### Preparation of Compounds

Compounds **1**, **2**, and **14** were prepared as previously reported.^13,16^

#### (*S*)-10-((5-Chloro-2-morpholinopyrimidin-4-yl)amino)-2-cyclopropyl-3,3-difluoro-7-methyl-1,2,3,4-tetrahydro-[1,4]oxazepino[2,3-*c*]quinolin-6(7H)-one (**3**)

A mixture of (S)-2-cyclopropyl-10-((2,5-dichloropyrimidin-4-yl)amino)-3,3-difluoro-7-methyl-1,2,3,4-tetrahydro-[1,4]oxazepino[2,3-*c*]quinolin-6(7H)-one (**2**) (7 mg, 0.014 mmol), morpholine (3 mg, 0.035 mmol), and DIPEA (12 μL, 0.071 mmol) in NMP (0.5 mL) was heated under microwave irradiation to 140 °C for 1 h. The resulting mixture was purified by reverse-phase chromatography eluting from 10–100% methanol in water (both modified with 0.1% formic acid), followed by further purification using an SCX-2 column to give **3** (4.1 mg, 0.0079 mmol, 56%). HRMS (ESI^+^): found 519.1720, expected 519.1717 for C_24_H_26_ClF_2_N_6_O_3_ [M + H]^+^; ^1^H NMR (600 MHz, CD_3_OD) δ 8.08 (d, *J* = 2.2 Hz, 1H), 7.98 (s, 1H), 7.91 (dd, *J* = 9.1, 2.2 Hz, 1H), 7.55 (d, *J* = 9.1 Hz, 1H), 4.52–4.36 (m, 2H), 3.71 (s, 3H), 3.69–3.66 (m, 4H), 3.65–3.62 (m, 4H), 3.35–3.28 (m, 1H), 1.44–1.37 (m, 1H), 0.83–0.75 (m, 1H), 0.70–0.64 (m, 1H), 0.64–0.58 (m, 1H), 0.38–0.32 (m, 1H).

#### (*S*)-10-((2-(3-Oxa-8-azabicyclo[3.2.1]octan-8-yl)-5-chloropyrimidin-4-yl)amino)-2-cyclopropyl-3,3-difluoro-7-methyl-1,2,3,4-tetrahydro-[1,4]oxazepino[2,3-*c*]quinolin-6(7*H*)-one (**4**)

The same method as for **3**, using 3-oxa-8-azabicyclo[3.2.1]octane hydrochloride and heating for 6 h. Purification by reverse-phase chromatography eluting from 10–100% methanol in water (both modified with 0.1% formic acid), followed by further purification using an SCX-2 column to give **4** (4.4 mg, 0.0081 mmol, 53%). HRMS (ESI^+^): found 545.1880, expected 545.1874 for C_26_H_28_ClF_2_N_6_O_3_ [M + H]^+^; ^1^H NMR (600 MHz, CD_3_OD) δ 8.03 (d, *J* = 2.1 Hz, 1H), 7.97 (s, 1H), 7.93 (dd, *J* = 9.1, 2.1 Hz, 1H), 7.54 (d, *J* = 9.1 Hz, 1H), 4.52–4.36 (m, 4H), 3.74–3.70 (m, 5 H), 3.58–3.54 (m, 2H), 3.34–3.27 (m, 1H), 2.04–1.97 (m, 2H), 1.97–1.91 (m, 2H), 1.43–1.35 (m, 1H), 0.82–0.76 (m, 1H), 0.69–0.63 (m, 1H), 0.63–0.57 (m, 1H), 0.38–0.32 (m, 1H).

#### (*S*)-10-((2-(8-Oxa-3-azabicyclo[3.2.1]octan-3-yl)-5-chloropyrimidin-4-yl)amino)-2-cyclopropyl-3,3-difluoro-7-methyl-1,2,3,4-tetrahydro-[1,4]oxazepino[2,3-*c*]quinolin-6(7*H*)-one (**5**)

The same method as for **3**, using 8-oxa-3-azabicyclo[3.2.1]octane hydrochloride and heating for 2 h. Purification by reverse-phase chromatography eluting from 10 to 100% methanol in water (both modified with 0.1% formic acid), followed by further purification using an SCX-2 column to give **6** (4.9 mg, 0.0072 mmol, 49%). HRMS (ESI^+^): found 545.1874, expected 545.1874 for C_26_H_28_ClF_2_N_6_O_3_ [M + H]^+^; ^1^H NMR (600 MHz, CD_3_OD) δ 8.08 (d, *J* = 2.2 Hz, 1H), 7.95 (s, 1H), 7.91 (dd, *J* = 9.1, 2.2 Hz, 1H), 7.54 (d, *J* = 9.1 Hz, 1H), 4.53–4.39 (m, 2H), 4.39–4.35 (m, 2H), 4.09–4.03 (m, 2H), 3.71 (s, 3H), 3.35–3.28 (m, 1H), 3.10–3.05 (m, 2H), 1.92–1.85 (m, 2H), 1.78–1.72 (m, 2H), 1.43–1.36 (m, 1H), 0.83–0.76 (m, 1H), 0.70–0.64 (m, 1H), 0.63–0.57 (m, 1H), 0.39–0.33 (m, 1H).

#### Step 1: *tert*-Butyl 3-acetyl-3,8-diazabicyclo[3.2.1]octane-8-carboxylate (**6a**)

Acetyl chloride (0.11 mL, 1.55 mmol) was added dropwise to a stirred solution of *tert*-butyl 3,8-diazabicyclo[3.2.1]octane-8-carboxylate (0.3 g, 1.41 mmol) and triethylamine (0.43 mL, 3.09 mmol) in anhydrous dichloromethane (4 mL) at 0 °C under argon. The reaction mixture was stirred at rt for 6 h, then concentrated *in vacuo*. The residue was redissolved in EtOAc (30 mL) and washed with 1 M HCl (2 × 15 mL), saturated aq. NaHCO_3_ (30 mL), and brine (30 mL). The organic layer was dried (Na_2_SO_4_) and concentrated *in vacuo*, affording the title compound (266 mg, 74%) as a yellow oil that was used without further purification. ^1^H NMR (500 MHz, CDCl_3_) δ 4.36–4.16 (m, 3H), 3.50–3.45 (m, 1H), 3.40 (br s, 1H), 2.85 (br s, 1H), 2.09 (s, 3H), 2.00–1.86 (m, 2H), 1.73–1.60 (m, 2H), 1.48 (s, 9H).

#### Step 2: 1-(3,8-Diazabicyclo[3.2.1]octan-3-yl)ethan-1-one Hydrochloride (**6b**)

4 M HCl in 1,4-dioxane (3.50 mL, 14 mmol) was added dropwise to a solution of **7a** (266 mg, 1.05 mmol) in DCM (5 mL) at 0 °C. The reaction mixture was allowed to warm to rt and stirred for 15 h, then concentrated *in vacuo* and dried under vacuum, affording the title compound (216 mg, 108%, 1.1328 mmol) as an off-white hygroscopic solid. ^1^H NMR (500 MHz, DMSO-*d*_6_) δ 4.17 (app d, *J* = 14.1 Hz, 1H), 4.00 (br s, 2H), 3.75–3.69 (m, 1H), 3.56–3.50 (m, 1H), 3.00 (app d, *J* = 14.1 Hz, 1H), 2.03 (s, 3H), 1.95–1.79 (m, 3H), 1.65–1.53 (m, 1H)

#### Step 3: (2*S*)-10-((2-(8-Acetyl-3,8-diazabicyclo[3.2.1]octan-3-yl)-5-chloropyrimidin-4-yl)amino)-2-cyclopropyl-3,3-difluoro-7-methyl-1,2,3,4-tetrahydro-[1,4]oxazepino[2,3-*c*]quinolin-6(7*H*)-one (**6**) and (2*S*)-10-((2-(3-acetyl-3,8-diazabicyclo[3.2.1]octan-8-yl)-5-chloropyrimidin-4-yl)amino)-2-cyclopropyl-3,3-difluoro-7-methyl-1,2,3,4-tetrahydro-[1,4]oxazepino[2,3-*c*]quinolin-6(7*H*)-one (**7**)

A mixture of **2** (14.8 mg, 0.0316 mmol), **7b** (12.8 mg, 0.067 mmol), and DIPEA (44 μL, 0.26 mmol) in NMP under argon was heated at 140 °C for 16 h. The reaction mixture was dissolved in DMSO (0.8 mL) and purified by reverse-phase chromatography (Biotage 12 g Ultra C 18 column; 10–60–80–100% methanol in water (0.1% formic acid modifier)). Fractions containing the product were combined and passed through an SCX-2 (2 g) column, washing with methanol (10 mL) and eluting with 2 M methanolic ammonia (30 mL). The ammonia fractions were combined and evaporated under reduced pressure to give a mixture of regioisomers. This was dissolved in a 1:1 mixture of DMSO/MeCN (1 mL) and purified by HPLC (2 injections; Phenomenex Gemini C18 110A column (5 μM, 250 × 10 mm^2^); 15 min gradient of 45:55 to 30:70 H_2_O/MeOH (both modified with 0.1% formic acid); flow rate 5 mL min^–1^; 1260 Infinity IIMS-Prep LC). The earlier eluting major product, example **6**, was obtained as a white solid (6.2 mg, 0.011 mmol). The later eluting minor product, example **7**, presumably resulting from the acetyl group moving to the less hindered position in the starting material, was obtained as an off-white solid (2.5 mg, 0.004 mmol).

#### 6

HRMS (ESI^+^): found 586.2120, expected 586.2139 for C_28_H_31_ClF_2_N_7_O_3_ [M + H]^+^; ^1^H NMR (600 MHz, CD_3_OD) δ 8.06–8.02 (m, 1H), 8.02–7.99 (m, 1H), 7.95 (ddd, *J* = 9.1, 4.6, 2.3 Hz, 1H), 7.57 (d, *J* = 9.1 Hz, 1H), 4.63-4.56 (m, 2H), 4.53–4.38 (m, 2H), 4.23–4.16 (m, 1H), 3.72 (s, 3H), 3.67–3.61 (m, 1H), 3.43 (d, *J* = 12.5 Hz, 1H), 3.36–3.27 (m, 1H), 2.92 (d, *J* = 13.0 Hz, 1H), 2.07 (s, 3H), 2.03–1.92 (m, 2H), 1.83–1.76 (m, 1H), 1.71–1.65 (m, 1H), 1.46–1.35 (m, 1H), 0.82–0.75 (m, 1H), 0.69–0.63 (m, 1H), 0.63–0.57 (m, 1H), 0.38–0.31 (m, 1H).

#### 7

HRMS (ESI^+^): found 586.2132, expected 586.2139 for C_28_H_31_ClF_2_N_7_O_3_ [M + H]^+^; ^1^H NMR (600 MHz, CD_3_OD) δ 8.07 (dd, *J* = 7.4, 2.3 Hz, 1H), 7.98 (d, *J* = 1.3 Hz, 1H), 7.92 (dd, *J* = 9.1, 2.3 Hz, 1H), 7.57 (d, *J* = 9.1 Hz, 1H), 4.71–4.63 (m, 1H), 4.54–4.38 (m, 2H), 4.36–4.24 (m, 3H), 3.73 (s, 3H), 3.36–3.27 (m, 1H), 3.08–2.98 (m, 2H), 2.11 (d, *J* = 1.4 Hz, 3H), 2.03–1.95 (m, 1H), 1.89–1.81 (m, 1H), 1.81–1.75 (m, 1H), 1.74–1.68 (m, 1H), 1.47–1.35 (m, 1H), 0.83–0.76 (m, 1H), 0.69–0.58 (m, 2H), 0.38–0.32 (m, 1H).

#### Step 1: 2,5-Dichloro-4-(methylthio)pyrimidine (**8a**)

2,4,5-trichloropyrimidine (3.27 mL, 28.54 mmol) was dissolved in THF (29 mL) and water (29 mL) and chilled to 0 °C. To this mixture was added sodium thiomethoxide (2.00 g, 28.54 mmol), and the reaction mixture was allowed to warm to room temperature and stirred for 4 h. EtOAc (50 mL) and water (50 mL) were added, and the layers separated. The aqueous layer was extracted with a further 50 mL of EtOAc, and the organic layers were combined, dried, and concentrated to afford a clear oil, which rapidly crystallized to give 2,5-dichloro-4-methylsulfanyl-pyrimidine (5.5 g, 99%) as a white solid. LCMS (ESI^+^): RT 1.35 min; *m*/*z* 194.9542 [M + H]^+^.

#### Step 2: (*1R,5S,7S*)-9-(5-Chloro-4-(methylthio)pyrimidin-2-yl)-3-oxa-9-azabicyclo[3.3.1]nonan-7-ol (**8b**)

An oven-dried microwave vial (2.0–5.0 mL volume) was charged with **8a** (234 mg, 1.20 mmol), *endo*-7-hydroxy-3-oxa-9-azabicyclo[3.3.1]nonane hydrochloride (238 mg, 1.32 mmol), and DIPEA (0.84 mL, 4.82 mmol). Isopropanol (3.4 mL) was added, the reaction vial was sealed with a cap, and the reaction mixture was heated at 120 °C in a heating block for 24 h. The reaction mixture was cooled to rt and concentrated *in vacuo*. Purification by flash chromatography (0–70% EtOAc in cyclohexane) afforded **8b** (221 mg, 61%) as a colorless oil, which solidified to an off-white solid when stored at 4 °C. LCMS (ESI^+^): RT 1.49 min; *m*/*z* 284.0726 [M – H_2_O + H]^+^; ^1^H NMR (500 MHz, CDCl_3_) δ 7.97 (s, 1H), 5.63 (d, *J* = 12.6 Hz, 1H), 4.80–4.65 (m, 2H), 4.00–3.92 (m, 3H), 3.87–3.82 (m, 2H), 2.48 (s, 3H), 2.26–2.15 (m, 2H), 1.89 (d, *J* = 15.0 Hz, 2H);

#### Step 3: (*1R,5S,7S*)-9-(5-Chloro-4-(methylsulfinyl)pyrimidin-2-yl)-3-oxa-9-azabicyclo-[3.3.1]-nonan-7-ol (**8c**)

**8b** (221 mg, 0.73 mmol) was dissolved in CH_2_Cl_2_ (3.7 mL) and MeCN (3.7 mL). 3-Chloroperoxybenzoic acid (409 mg, 1.82 mmol) was added, and the reaction mixture was stirred for 3 h at room temperature. CH_2_Cl_2_ (30 mL) was added, and the reaction mixture was washed with 10% aq. Na_2_SO_3_ (30 mL) and with saturated aq. NaHCO_3_ (20 mL). The aqueous layer was extracted with CH_2_Cl_2_ (20 mL), and the organic layers were combined and washed with brine, dried (MgSO_4_), and concentrated *in vacuo*. Purification by flash chromatography (0–10% MeOH in CH_2_Cl_2_) afforded the title compound (214 mg, 88%) as a pale yellow solid. LCMS (ESI^+^): RT 1.00 min; *m*/*z* 316.0537 [M – H_2_O + H]^+^; ^1^H NMR (500 MHz, CD_3_OD) δ 8.59 (s, 1H), 4.83–4.77 (m, 1H), 4.69–4.64 (m, 1H), 4.01–3.99 (m, 1H), 3.99–3.96 (m, 1H), 3.94–3.89 (m, 1H), 3.87–3.80 (m, 2H), 3.35 (s, 3H), 2.30–2.18 (m, 2H), 1.94–1.86 (m, 2H).

#### Step 4: (*S*)-10-((5-Chloro-2-((1*R*,5*S*,7*R*)-7-hydroxy-3-oxa-9-azabicyclo[3.3.1]-nonan-9-yl)pyrimidin-4-yl)amino)-2-cyclopropyl-3,3-difluoro-7-methyl-1,2,3,4-tetrahydro-[1,4]oxazepino[2,3-*c*]quinolin-6(7*H*)-one (**8**)

A microwave vial (2–5 mL volume) was charged with (*S*)-10-amino-2-cyclopropyl-3,3-difluoro-7-methyl-1,2,3,4-tetrahydro-[1,4]oxazepino[2,3-*c*]quinolin-6(7*H*)-one (**1**, 29 mg, 0.09 mmol) and **8c** (36 mg, 0.11 mmol). Trifluoroethanol (1.0 mL) was added, followed by trifluoroacetic acid (7.7 μL, 0.10 mmol). The reaction vial was flushed with Ar and sealed with a cap. The reaction mixture was heated at 70 °C in a heating block for 20 h. The reaction mixture was cooled to rt and concentrated *in vacuo*. The residue was redissolved in DMSO (1 mL) and directly purified by reverse-phase chromatography (Biotage reverse-phase 12 g C 18 column; 10–100% MeOH in H_2_O (containing 0.1% formic acid)). The product-containing fractions were passed through an SCX-2 (2 g) column, eluting with MeOH (15 mL) followed by 2 N methanolic ammonia (30 mL). The basic fraction was concentrated *in vacuo*, affording the title compound (16 mg, 31%) as an off-white solid. HRMS (ESI^+^): found 575.1987, expected 575.1985 for C_27_H_30_ClF_2_N_6_O_4_^+^ [M + H]^+^; ^1^H NMR (600 MHz, CD_3_OD) δ 8.02–7.99 (m, 2H), 7.88 (dd, *J* = 9.1, 1.7 Hz, 1H), 7.54 (d, *J* = 9.1 Hz, 1H), 4.62–4.37 (m, 4H), 3.94–3.84 (m, 3H), 3.79–3.73 (m, 2H), 3.71 (s, 3H), 3.30–3.26 (m, 1H), 2.24–2.14 (m, 2H), 1.83–1.73 (m, 2H), 1.43–1.36 (m, 1H), 0.82–0.76 (m, 1H), 0.69–0.63 (m, 1H), 0.63–0.57 (m, 1H), 0.37–0.31 (m, 1H).

#### (*S*)-10-((2-(Azetidin-1-yl)-5-chloropyrimidin-4-yl)amino)-2-cyclopropyl-3,3-difluoro-7-methyl-1,2,3,4-tetrahydro-[1,4]oxazepino[2,3-*c*]quinolin-6(7*H*)-one (**9**)

The same method as for **3**, using azetidine and heating at 80 °C for 1 h. Purification by reverse-phase chromatography eluting from 10–100% methanol in water (both modified with 0.1% formic acid), followed by further purification using an SCX-2 column to give **9** (7.5 mg, 0.015 mmol, 53%). HRMS (ESI^+^): found 489.1602, expected 489.1617 for C_23_H_24_ClF_2_N_6_O_2_^+^ [M + H]^+^; ^1^H NMR (600 MHz, CD_3_OD) δ 8.17 (d, *J* = 2.3 Hz, 1H), 8.02 (dd, *J* = 9.1, 2.3 Hz, 1H), 7.90 (s, 1H), 7.50 (d, *J* = 9.1 Hz, 1H), 4.54–4.34 (m, 2H), 4.10–4.03 (m, 4H), 3.69 (s, 3H), 3.37–3.28 (m, 1H), 2.32 (quin, *J* = 7.5 Hz, 2H), 1.42–1.34 (m, 1H), 0.83–0.77 (m, 1H), 0.71–0.64 (m, 1H), 0.61–0.58 (m, 1H), 0.40-0.33 (m, 1H).

#### (*S*)-10-((5-Chloro-2-(3-methoxyazetidin-1-yl)pyrimidin-4-yl)amino)-2-cyclopropyl-3,3-difluoro-7-methyl-1,2,3,4-tetrahydro-[1,4]oxazepino[2,3-*c*]quinolin-6(7*H*)-one (**10**)

The same method as for **3**, using 3-methoxyazetidine hydrochloride and heating at 80 °C for 1 h. Purification by reverse-phase chromatography eluting from 10 to 100% methanol in water (both modified with 0.1% formic acid), followed by further purification using an SCX-2 column to give **10** (7.7 mg, 0.015 mmol, 53%). HRMS (ESI^+^): found 519.1708, expected 519.1723 for C_24_H_26_ClF_2_N_6_O_3_^+^ [M + H]^+^; ^1^H NMR (600 MHz, CD_3_OD) δ 8.18 (d, *J* = 2.3 Hz, 1H), 8.00 (dd, *J* = 9.1, 2.3 Hz, 1H), 7.93 (s, 1H), 7.52 (d, *J* = 9.1 Hz, 1H), 4.52–4.37 (m, 2H), 4.31-4.26 (m, 1H), 4.25–4.19 (m, 2H), 3.90–3.86 (m, 2H), 3.71 (s, 3H), 3.36–3.28 (m, 4H), 1.43–1.35 (m, 1H), 0.83–0.76 (m, 1H), 0.72–0.66 (m, 1H), 0.64–0.59 (m, 1H), 0.40-0.34 (m, 1H).

#### (*S*)-10-((5-Chloro-2-(3,3-difluoroazetidin-1-yl)pyrimidin-4-yl)amino)-2-cyclopropyl-3,3-difluoro-7-methyl-1,2,3,4-tetrahydro-[1,4]oxazepino[2,3-*c*]quinolin-6(7*H*)-one (**11**)

The same method as for **3**, using 3,3-difluoroazetidine hydrochloride and heating at 120 °C for 19 h. Purification by reverse-phase chromatography eluting from 10–100% methanol in water (both modified with 0.1% formic acid), followed by further purification using an SCX-2 column to give **11** (4.3 mg, 0.008 mmol, 53%). HRMS (ESI^+^): found 525.1412, expected 525.1429 for C_23_H_22_ClF_4_N_6_O_2_^+^ [M + H]^+^; ^1^H NMR (600 MHz, CD_3_OD) δ 8.14 (d, *J* = 2.1 Hz, 1H), 8.02 (s, 1H), 7.95 (dd, *J* = 9.1, 2.1 Hz, 1H), 7.55 (d, *J* = 9.1 Hz, 1H), 4.52–4.34 (m, 6 H), 3.72 (s, 3H), 3.36–3.29 (m, 1H), 1.44–1.37 (m, 1H), 0.83–0.76 (m, 1H), 0.71–0.65 (m, 1H), 0.65–0.59 (m, 1H), 0.39–0.33 (m, 1H).

#### (*S*)-1-(5-Chloro-4-((2-cyclopropyl-3,3-difluoro-7-methyl-6-oxo-1,2,3,4,6,7-hexahydro-[1,4]oxazepino[2,3-*c*]quinolin-10-yl)amino)pyrimidin-2-yl)azetidine-3-carbonitrile (**12**)

The same method as for **3**, using azetidine-3-carbonitrile hydrochloride and heating at 80°C for 1.5 h. Purification by reverse-phase chromatography eluting from 40–90% methanol in water (both modified with 0.1% formic acid), followed by further purification using an SCX-2 column to give **12** (11.2 mg, 0.022 mmol, 84%). HRMS (ESI^+^): found 514.1570, expected 514.1570 for C_24_H_23_ClF_2_N_7_O_2_^+^ [M + H]^+^; ^1^H NMR (600 MHz, CD_3_OD) δ 8.15 (d, *J* = 2.3 Hz, 1H), 8.01–7.97 (m, 2H), 7.55 (d, *J* = 9.2 Hz, 1H), 4.54–4.38 (m, 2H), 4.35 (q, *J* = 8.6 Hz, 2H), 4.22–4.19 (m, 2H), 3.73 (dd, *J* = 8.4, 2.5 Hz, 1H), 3.72 (s, 3H), 3.42–3.28 (m, 1H), 1.48–1.38 (m, 1H), 0.86–0.78 (m, 1H), 0.75–0.68 (m, 1H), 0.66–0.60 (m, 1H), 0.47–0.32 (m, 1H).

#### (*S*)-10-((5-Chloro-2-(3-fluoroazetidin-1-yl)pyrimidin-4-yl)amino)-2-cyclopropyl-3,3-difluoro-7-methyl-1,2,3,4-tetrahydro-[1,4]oxazepino[2,3-*c*]quinolin-6(7*H*)-one (**13**)

The same method as for **3**, using 3-fluoroazetidine hydrochloride and heating at 80°C for 1.5 h. Purification by reverse-phase chromatography eluting from 40–90% methanol in water (both modified with 0.1% formic acid), followed by further purification using an SCX-2 column to give **13** (6.9 mg, 0.014 mmol, 54%). HRMS (ESI^+^): found 507.1519, expected 507.1518 for C_23_H_23_ClF_3_N_6_O_2_^+^ [M + H]^+^; ^1^H NMR (600 MHz, CD_3_OD) δ 8.16 (d, *J* = 2.3 Hz, 1H), 8.00 (dd, *J* = 9.1, 2.3 Hz, 1H), 7.97 (s, 1H), 7.54 (d, *J* = 9.2 Hz, 1H), 5.39 (dtt, *J* = 57.2, 6.1, 3.2 Hz, 1H), 4.54–4.29 (m, 4H), 4.16–4.05 (m, 2H), 3.72 (s, 3H), 3.42–3.25 (m, 1H), 1.48–1.36 (m, 1H), 0.85–0.77 (m, 1H), 0.74–0.66 (m, 1H), 0.66–0.58 (m, 1H), 0.45–0.32 (m, 1H).

#### (*S*)-2-Cyclopropyl-10-((2,3-dichloropyridin-4-yl)amino)-3,3-difluoro-7-methyl-1,2,3,4-tetrahydro-[1,4]oxazepino[2,3-*c*]quinolin-6(7*H*)-one (**15**)

A mixture of (2*S*)-10-amino-2-cyclopropyl-3,3-difluoro-7-methyl-2,4-dihydro-1H-[1,4]oxazepino[2,3-*c*]quinolin-6-one (**1**, 10.2 mg, 0.032 mmol), 2,3-dichloro-4-iodopyridine (10.3 mg, 0.038 mmol), Xantphos (11 mg, 0.020 mmol), Pd_2_(dba)_3_ (4 mg, 0.0039 mmol), and cesium carbonate (80 mg, 0.25 mmol) in toluene (0.3 mL) and DMF (0.3 mL) under argon was heated at 80 °C for 1 h. Water (15 mL) was added, and the aqueous mixture was extracted with CH_2_Cl_2_ (4 ×10 mL). The organic extracts were combined, washed with brine (10 mL), dried (Na_2_SO_4_), and stirred overnight in the presence of MP-TMT to remove residual Pd. The beads were filtered off and washed with CH_2_Cl_2_, and the filtrate was concentrated under reduced pressure. The crude product was purified by reverse-phase chromatography eluting from 10–100% methanol in water (both modified with 0.1% formic acid), affording the title compound as the formate salt (7.4 mg, 0.016 mmol, 50%). HRMS (ESI^+^): found 467.0853, expected 467.0853 for C_21_H_19_Cl_2_F_2_N_4_O_2_^+^ [M + H]; ^1^H NMR (600 MHz, CD_3_OD) δ 8.00 (d, *J* = 2.3 Hz, 1H), 7.83 (d, *J* = 5.8 Hz, 1H), 7.63 (d, *J* = 9.0 Hz, 1H), 7.54 (dd, *J* = 9.0, 2.3 Hz, 1H), 6.74 (d, *J* = 5.8 Hz, 1H), 4.53–4.38 (m, 2H), 3.73 (s, 3H), 3.32–3.24 (m, 1H), 1.42–1.35 (m, 1H), 0.80–0.74 (m, 1H) 0.66–0.55 (m, 2H), 0.35–0.28 (m, 1H).

#### (*S*)-10-((3-chloro-2-methylpyridin-4-yl)amino)-2-cyclopropyl-3,3-difluoro-7-methyl-1,2,3,4-tetrahydro-[1,4]oxazepino[2,3-*c*]quinolin-6(7*H*)-one (**16**)

The same method as for **15**, using 3,4-dichloro-2-methylpyridine with heating at 100 °C for 1 h. Purification by reverse-phase chromatography eluting from 10–100% methanol in water (both modified with 0.1% formic acid). The product was further purified by flash column chromatography (0–10% MeOH in DCM) to give **16** (7.4 mg, 0.017 mmol, 35%). HRMS (ESI^+^): found 447.1384, expected 447.1399 for C_22_H_22_ClF_2_N_4_O_2_^+^ [M + H]; ^1^H NMR (600 MHz, CD_3_OD) δ 7.96 (d, *J* = 2.3 Hz, 1H), 7.90 (d, *J* = 5.9 Hz, 1H), 7.60 (d, *J* = 9.0 Hz, 1H), 7.53 (dd, *J* = 9.0, 2.3 Hz, 1H), 6.70 (d, *J* = 5.9 Hz, 1H), 4.52–4.37 (m, 2H), 3.72 (s, 3H), 3.28 (ddd, *J* = 18.6, 10.1, 5.2 Hz, 1H), 2.55 (s, 3H), 1.42–1.35 (m, 1H), 0.80–0.73 (m, 1H), 0.66–0.55 (m, 2H), 0.35–0.29 (m, 1H).

#### (*S*)-10-((3-chloro-2-fluoropyridin-4-yl)amino)-2-cyclopropyl-3,3-difluoro-7-methyl-1,2,3,4-tetrahydro-[1,4]oxazepino[2,3-*c*]quinolin-6(7*H*)-one (**17**, **CCT374705**)

A mixture of (2S)-10-amino-2-cyclopropyl-3,3-difluoro-7-methyl-2,4-dihydro-1H-[1,4]oxazepino[2,3-*c*]quinolin-6-one (**1**, 3.6 g, 11.2 mmol), 4-bromo-3-chloro-2-fluoropyridine (2.95 g, 14 mmol), Xantphos (972 mg, 1.68 mmol), Pd(OAc)_2_ (251 mg, 1.12 mmol), and cesium carbonate (5.14 g, 15.7 mmol) in 1,4-dioxane (25 mL) under argon was heated at 100 °C for 50 min. The reaction mixture was concentrated under reduced pressure onto silica and purified by flash column chromatography (50–100% EtOAc in cyclohexane, then 100% EtOAc). The resulting material was recrystallized from EtOAc to give the title compound (3.45 g, 68%, 7.65 mmol). HRMS (ESI^+^): found 451.1129, expected 451.1146 for C_21_H_19_ClF_3_N_4_O_2_^+^ [M + H]; ^1^H NMR (600 MHz, CD_3_OD) δ 8.02 (d, *J* = 2.3 Hz, 1H), 7.70 (d, *J* = 5.9 Hz, 1H), 7.65 (d, *J* = 9.0 Hz, 1H), 7.56 (dd, *J* = 8.9, 2.4 Hz, 1H), 6.71 (d, *J* = 5.9 Hz, 1H), 4.55–4.40 (m, 2H), 3.76 (s, 3H), 3.32–3.26 (m, 1H), 1.45–1.36 (m, 1H), 0.83–0.75 (m, 1H), 0.68–0.58 (m, 2H), 0.37–0.30 (m, 1H).

#### (*S*)-10-((2-chloro-3-fluoropyridin-4-yl)amino)-2-cyclopropyl-3,3-difluoro-7-methyl-1,2,3,4-tetrahydro-[1,4]oxazepino[2,3-*c*]quinolin-6(7*H*)-one (**18**)

The same method as for **16**, using 2-chloro-3-fluoro-4-iodopyridine with heating for 1.5 h. Purification by reverse-phase chromatography eluting from 10–100% methanol in water (both modified with 0.1% formic acid) to give **18** as the formate salt (7.3 mg, 0.016 mmol, 47%). HRMS (ESI^+^): found 451.1144, expected 451.1146 for C_21_H_19_ClF_3_N_4_O_2_^+^ [M + H]; ^1^H NMR (600 MHz, CD_3_OD) δ 7.95 (d, *J* = 2.3 Hz, 1H), 7.78 (d, *J* = 5.7 Hz, 1H), 7.60 (d, *J* = 9.0 Hz, 1H), 7.52 (dd, *J* = 9.0, 2.3 Hz, 1H), 6.94 (app. t, *J* = 6.0 Hz, 1H), 4.53–4.37 (m, 2H), 3.72 (s, 3H), 3.28 (ddd, *J* = 18.7, 10.1, 5.2 Hz, 1H), 1.43–1.35 (m, 1H), 0.81–0.74 (m, 1H) 0.66–0.55 (m, 2H), 0.36–0.30 (m, 1H).

#### (*S*,*E*)-*N*-(cyclopropylmethylene)-2-methylpropane-2-sulfinamide (**19a**)

To a solution of cyclopropanecarboxaldehyde (24.66 mL, 330.03 mmol) in anhydrous DCM (100 mL) was added MgSO_4_ (59.59 g, 495.05 mmol), pyridinium *p*-toluenesulfonate (2.07 g, 8.25 mmol), and (S)-(−)-2-methyl-2-propanesulfinamide (20 g, 165.02 mol). The reaction was stirred at rt for 18 h. The reaction mixture was filtered and washed with DCM (10 mL). The solvent was removed under reduced pressure, and the residue was purified by flash column chromatography (Sfar 100 g, 0–50% EtOAc in cyclohexane), affording the title compound (26.31 g, 92%) as a colorless liquid/oil. LCMS (ESI^+^): RT 1.24 min; *m*/*z* 196.078 [M + Na]^+^; ^1^H NMR (500 MHz, CDCl_3_) δ 7.37 (d, *J* = 7.8 Hz, 1H), 1.97–1.81 (m, 1H), 1.09 (s, 9H), 1.06–0.96 (m, 2H), 0.91–0.76 (m, 2H).

#### Ethyl (*S*)-3-(((*S*)-*tert*-butylsulfinyl)amino)-3-cyclopropyl-2,2-difluoropropanoate (**19b**)

Zinc powder (325 mesh) (12.62 g, 193.0 mmol) was suspended in anhydrous THF (250 mL) in a two-necked 1 L RBF equipped with a reflux condenser under N_2_. To this was added ethyl bromodifluoroacetate (1.23 mL, 9.59 mmol). DIBAL-H (1 M in toluene) (5.82 mL, 5.82 mmol) was added, and the reaction was heated to 40 °C. Ethyl bromodifluoroacetate (23.38 mL, 182.4 mmol) was added over 20 min, and the reaction mixture was stirred at 40 °C for a further 15 min. After allowing the reaction mixture to cool to rt, the resulting solution was cannulated into a solution of **19a** (15.0 g, 86.57 mmol) in anhydrous THF (150 mL). The reaction mixture was stirred at rt under N_2_ for 1 h. The reaction mixture was cooled to 0 °C, and water (5 mL) was added dropwise. The reaction mixture was allowed to stir for 10 min, and then 1 M HCl (20 mL) was added. The solvent was concentrated under reduced pressure, and the mixture was extracted with EtOAc (2 ×100 mL). The organic layers were combined and washed with 1 M NaOH (2 ×30 mL) and brine (30 mL), dried with MgSO_4_, and the solvent was removed under reduced pressure, affording the title compound (27.0 g, >99%) as a yellow oil, which was used without further purification. LCMS (ESI^+^): RT 1.37 min; *m*/*z* 320.108 [M + Na]^+^; ^1^H NMR (600 MHz, CDCl_3_) δ 4.32 (qd, *J* = 7.2, 1.8 Hz, 2H), 3.84 (d, *J* = 7.1 Hz, 1H), 3.16–3.07 (m, 1H), 1.33 (t, *J* = 7.2 Hz, 3H), 1.19 (s, 9H), 1.03–0.96 (m, 1H), 0.73–0.67 (m, 1H), 0.67–0.61 (m, 1H), 0.47–0.41 (m, 1H), 0.41–0.36 (m, 1H). ^19^F NMR (471 MHz, CDCl_3_) δ −113.48 (dd, *J* = 259.1, 11.4 Hz), −114.55 (dd, *J* = 258.8, 11.6 Hz).

#### (*S*)-*N*-((*S*)-1-cyclopropyl-2,2-difluoro-3-hydroxypropyl)-2-methylpropane-2-sulfinamide (**19c**)

**19b** (15.56 g, 52.33 mmol) was dissolved in anhydrous MeOH (300 mL) and cooled to 0 °C. Sodium borohydride (5.00 g, 132.17 mmol) was added portionwise, and the reaction was allowed to warm to rt and stirred for 1 h. Upon completion, the reaction was cooled to 0 °C and quenched with the addition of saturated NH_4_Cl solution (5 mL). The mixture was concentrated under reduced pressure and then extracted with EtOAc (3 × 50 mL). The organic layers were combined and washed with water (2 × 20 mL) and brine (20 mL), and the solvent was removed under reduced pressure. The residue was purified by flash column chromatography (Sfar 100 g, 0–5% MeOH in DCM), affording the title compound as a colorless oil (5.43 g, 41% (across 2 steps)). LCMS (ESI^+^): RT 1.17 min; *m*/*z* 278.100 [M + Na]^+^; ^1^H NMR (600 MHz, CDCl_3_) δ 4.66 (td, *J* = 7.2, 1.8 Hz, 1H), 4.08 (d, *J* = 8.3 Hz, 1H), 3.97 (dddd, *J* = 21.8, 13.3, 7.4, 6.1 Hz, 1H), 3.85 (tdd, *J* = 12.8, 9.6, 7.2 Hz, 1H), 3.25–3.13 (m, 1H), 1.25 (s, 9H), 1.10 (tdd, *J* = 8.1, 5.0, 3.2 Hz, 1H), 0.71–0.55 (m, 2H), 0.48 (dq, *J* = 9.7, 5.0 Hz, 1H), 0.35 (dq, *J* = 10.0, 5.0 Hz, 1H). ^19^F NMR (471 MHz, CDCl_3_) δ −110.96 (ddt, *J* = 260.9, 21.7, 7.8 Hz), −118.72 (dddd, *J* = 260.8, 18.1, 12.4, 6.2 Hz).

#### (*S*)-3-amino-3-cyclopropyl-2,2-difluoropropan-1-ol Hydrochloride (**19**)

**19c** (5.40 g, 21.15 mmol) was dissolved in HCl (4 N in 1,4-dioxane) (36 mL, 144.0 mmol), and the reaction mixture was stirred for 1.5 h. The solvent was concentrated under reduced pressure, and Et_2_O (20 mL) was added to the residue. The mixture was sonicated at rt for 1 h, and the resulting solid was filtered and washed with Et_2_O (20 mL). The solid was dried under vacuum, affording the title compound (3.29 g, 83%) as an off-white solid. LCMS (ESI^+^): RT 0.16 min; *m*/*z* 152.086 [M + H]^+^; ^1^H NMR (500 MHz, CD_3_OD) δ 4.07–3.89 (m, 2H), 3.10 (ddd, *J* = 19.4, 10.7, 4.5 Hz, 1H), 1.16–1.04 (m, 1H), 0.91–0.77 (m, 2H), 0.77–0.68 (m, 1H), 0.62–0.53 (m, 1H). ^19^F NMR (471 MHz, CD_3_OD) δ −112.05 (dddd, *J* = 254.7, 24.2, 14.5, 4.7 Hz), −122.28 (ddt, *J* = 254.7, 19.3, 7.5 Hz).

## Supporting Information Available

Molecular formula strings with associated biochemical assay data and calculated properties (CSV)
Selectivity data for CCT374705 (XLSX)
Protein production, purification, and crystallography (BCL6 constructs used, methods for expression, purification, and crystallography, including data collection, processing, and refinement); biological assay conditions (methods for TR-FRET, MSD, and cell proliferation assays); physicochemical and *in vitro* DMPK assay conditions (methods for NMR and HPLC solubility, log *D*, microsomal clearance, caco-2, and protein binding). *In vivo* PK and PD experimental methods, including preparation of tumor xenografts. Further analytical data for compound **21** (CCT374705), including LCMS, chiral HPLC, and qNMR; summary statistics and individual replicate values of TR-FRET and NanoBRET assays (Tables S1 and S2); crystallographic data collection and refinement statistics (Table S3); composition of solution formulation for **CCT374705** (Table S4); qNMR purity analysis for **CCT374705** (Table S5); free mean mouse (BALB/c) blood concentrations (nM) of **9** after PO dosing at 5 mg/kg and extrapolated (assuming linear PK) to 50 mg/kg PO dosing (Figure S1); free mean mouse (BALB/c) blood concentrations (nM) of **4**, **8**, **11**, and **CCT374705** after PO dosing at 5 mg/kg (Figure S2); X-ray structure of the BCL6 BTB domain with bound ligand **CCT374705** (Figure S3); chiral HPLC analysis of **CCT374705** (Figure S4) (PDF)

## Accession Codes

Atomic coordinates and structure factors for the crystal structure of BCL6 with **CCT374705** can be accessed using PDB code 8C78. Authors will release the atomic coordinates and experimental data upon article publication.

## Abbreviations Used

APCI: atmospheric pressure chemical ionization
BCL6: B-cell lymphoma 6 protein
BCOR: BCL6 corepressor protein
BTB: broad-complex, tramtrack and bric a brac (domain), also known as POZ (Poxvirus and zinc finger) domain
CL: clearance
CL_int_: intrinsic clearance
CL_u_: unbound clearance
*c*_max_: maximum (peak) concentration achieved after a single dose
DCM: dichloromethane
DC_50_: concentration of compound at which 50% of the maximally observed protein degradation (*D*_max_) is achieved
DIPEA: *N,N*-diisopropylethylamine
DLBCL: diffuse large B-cell lymphoma
*D*_max_: maximal percentage degradation of protein achieved
EtOAc: ethyl acetate
GAPDH: glyceraldehyde 3-phosphate dehydrogenase
HAC: heavy atom count
MeCN: acetonitrile
MLM: mouse liver microsomes
MSD: mesoscale discovery, an assay method similar to ELISA, using electrochemiluminescence as a detection technique
NCOR: nuclear receptor corepressor 1
NMP: *N*-methyl-2-pyrrolidone
PFP: pentafluorophenyl
PPI: protein–protein interaction
PPTS: pyridinium *p*-toluenesulfonate
QToF: quadrupole time-of-flight
SCID: severe combined immunodeficient (mouse model)
SCX-2: SCX (strong cation exchange)-2 is a propylsulfonic acid bonded sorbent
SFC: supercritical fluid chromatography
SMRT: silencing mediator for retinoid or thyroid hormone receptor (also known as nuclear receptor corepressor 2 or NCOR2)
S_N_Ar: nucleophilic aromatic substitution
sol.: solubility
STR: short tandem repeat (analysis)
TR-FRET: time-resolved fluorescence energy transfer
