# Supplementary material for: Discovery of an In Vivo Chemical Probe for BCL6 Inhibition by Optimization of Tricyclic Quinolinones
Source: J Med Chem. 2023 Apr 7;66(8):5892–906. doi: 10.1021/acs.jmedchem.3c00155 (PMC10150366; doi:10.1021/acs.jmedchem.3c00155)
Supplement: Supplementary file 3 — jm3c00155_si_003.pdf [file jm3c00155_si_003.pdf]

Supplementary information for:

Discovery of an *In Vivo* Chemical Probe for BCL6 Inhibition by Optimisation of Tricyclic Quinolinones

Alice C. Harnden[†,‖], Owen A. Davis[†,‖], Gary M. Box[†], Angela Hayes[†], Louise D. Johnson[†], Alan T. Henley[†], Alexis K. de Haven Brandon[†], Melanie Valenti[†], Kwai-Ming J. Cheung[†], Alfie Brennan[†], Rosemary Huckvale[†], Olivier A. Pierrat[†], Rachel Talbot[†], Michael D. Bright[†], Hafize Aysin Akpinar[†], Daniel S. J. Miller[†], Dalia Tarantino[†], Sharon Gowan[†], Selby de Klerk[†], Peter Craig McAndrew[†], Yann-Vaï Le Bihan[†,#], Mirco Meniconi[†], Rosemary Burke[†], Vladimir Kirkin[†], Rob L. M. van Montfort[†,#], Florence I. Raynaud[†], Olivia W. Rossanese[†], Benjamin R. Bellenie[†,*], and Swen Hoelder[†,*]

Author Address

[†]Cancer Research UK Cancer Therapeutics Unit and [#]Division of Structural Biology, The Institute of Cancer Research, London SM2 5NG, U.K.

*E-mail: Benjamin.Bellenie@icr.ac.uk. Phone: +44 (0) 2087224602 (B.B.).

*E-mail: Swen.Hoelder@icr.ac.uk. Phone: +44 (0) 2087224353 (S.H.)

Contents

1. **Supplementary experimental: protein production, purification and crystallography** 5
    - 1.1 **BCL6 constructs used for assays and crystallography** 5
    - 1.2 **BCL6 expression** 5
    - 1.3 **BCL6 purification** 5
    - 1.4 **BCL6 crystallisation** 6
    - 1.5 **Crystallographic data collection, processing and refinement** 6
2. **Supplementary experimental: biological assay conditions** 6
    - 2.1 **TR-FRET assay** 6
    - 2.2 **NanoBRET Assay** 7
    - 2.3 **Cell proliferation assay** 8
3. **Supplementary experimental: physicochemical assays** 9
    - 3.1 **NMR solubility assay** 9
    - 3.2 **$logD_{7.4}$ assay** 9
4. **Supplementary experimental: in vitro DMPK assays** 10
    - 4.1 **Microsomal clearance assay** 10
    - 4.2 **Caco-2 assay** 10
    - 4.3 **Protein binding assay** 11
5. **Supplementary experimental: in vivo PK and PD experiments** 12
    - 5.1 ***In vivo* pharmacokinetic studies** 12
    - 5.2 **Formulation of CCT374705 (17)** 13
    - 5.3 ***In vivo* pharmacodynamic (PK/PD) and efficacy studies** 13
        - *5.3.1 Preparation of Karpas 422 Cells* 13
        - *5.3.2 Establishing a Karpas 422 subline* 13
        - *5.3.3 Preparation of Karpas 422 xenograft model in mouse* 14
        - *5.3.4 Tissue sampling* 14
        - 5.3.5 *Tumour lysis and Wes method* 15
        - 5.3.6 *Quantitative RT-PCR* 15
6. **Analytical data for CCT374705** 16
    - 6.1 **LCMS method** 16
    - 6.2 **HPLC and LCMS traces for CCT374705** 16
    - 6.3 **qNMR method** 16
    - 6.4 **qNMR analysis for CCT374705** 17
7. **Supplementary Tables** 18

Supplementary Table S1: 18
Supplementary Table S2: 19
Supplementary Table S3: 20
Supplementary Table S4: 21
Supplementary Table S5: 21

8. **Supplementary Figures** 22

Supplementary Figure S1: 22
Supplementary Figure S2: 23
Supplementary Figure S3: 24
Supplementary Figure S4: 24

9. **References for supplementary information** 25

1. Supplementary experimental: protein production, purification and crystallography

1.1 BCL6 constructs used for assays and crystallography

A first construct of BCL6 BTB domain, which we will refer to as Trx-6His-HRV3C-BCL6, was obtained by sub-cloning the sequence coding for residues 5-129 of human BCL6, corresponding to its BTB domain, into a pET48b vector with N-terminal Thioredoxin and 6-Histidine tags, followed by a HRV-3C protease cleavage site. For the TR-FRET assay, the Trx-6His-HRV3C-BCL6 protein construct was used without cleaving the tag, as a 6His was needed to bind to the anti-6His-Terbium antibody.

For crystallography with compound **17** (**CCT374705**), the construct described above was modified to introduce a Flag Tag and a TEV cleavage site between the HRV3C and BCL6 sequences. This construct will be referred to as Flag-TEV-BCL6.

1.2 BCL6 expression

For both plasmid constructs described above, transformed BL21-AI *E. coli* cells were grown in LB media supplemented with 50 mg/L kanamycin at 37 ^{o}C until an $OD_{600\ nm}$ of 0.6 was reached. Protein expression was then induced by addition of 0.2 mM IPTG and 0.2 % Arabinose. Expression was carried out at 18 ^{o}C for 18 hours. Cells were harvested by centrifugation (5500 g for 30 minutes at 4 ^{o}C) and stored at -80 ^{o}C.

1.3 BCL6 purification

Cells were re-suspended in a buffer composed of 20 mM Tris pH 8, 250 mM NaCl, 1 mM $MgCl_2$, 0.5 mM TCEP and 5 % glycerol, 1x cOmplete^{TM} ULTRA protease inhibitors and 12.5 U/ml Benzonaze. Cells were lysed by sonication followed by centrifugation at 21,000 g for 45 minutes at 4 ^{o}C. The supernatant was loaded onto a HisTrap FF column followed by on-column cleavage of the Trx-6His-HRV3C tag by addition of 2 mg of HRV-3C protease. The cleaved Flag-TEV-BCL6 5-129 was then eluted and purified further by ResourceQ and gel filtration using a HiLoad 26/60 Superdex75 column in a buffer containing 20 mM HEPES pH 7.5, 250 mM NaCl, 1 mM TCEP and 5 % glycerol. The final protein was assessed for purity and molar mass by SDS-PAGE and high-resolution mass spectrometry, respectively.

For the uncleaved Trx-6His-HRV3C-BCL6 protein construct to be used in TR-FRET, the protein was directly eluted from the HisTrap FF column without HRV-3C treatment, and submitted to Superdex75 gel filtration as described above.

1.4 BCL6 crystallisation

The purified Flag-TEV-BCL6 5-129 was concentrated to a final protein concentration of 11 mg/mL using a centrifugal concentrator with a 10 kDa molecular weight cut-off. Crystals were grown at 18 ^{o}C in hanging drops composed of 1.5 µL of the Flag-TEV-BCL6 complex plus 1.5 µL of a crystallisation solution consisting of 0.1 M Tris pH 7.5 and 0.80 M Na/K Tartrate, against 300 µL of crystallisation solution. Crystals typically grew in 2 days, and compound **CCT374705** was soaked into crystals by addition of 0.2 µL (dissolved in DMSO to a final concentration of 50 mM) directly to crystallisation drops, followed by 24 hours incubation. Crystals were then cryo-protected in a solution composed of the crystallisation reagent supplemented with 30 % ethylene glycol and cryo-cooled in liquid nitrogen.

1.5 Crystallographic data collection, processing and refinement

X-ray data were collected at Diamond Light Source, Harwell campus, Oxfordshire, UK, on beamlines I03. The chosen crystal belonged to the space group P 6_1 2 2 and diffracted to 1.8 Å resolution. Dataset was integrated with DIALS[1] and scaled and merged with AIMLESS[2]. Structure was solved by molecular replacement using PHASER[3] with a publicly available BCL6 structure[4] (PDB code 3BIM) with ligand and water molecules removed used as molecular replacement model. The protein/ligand structure was manually corrected and rebuilt in COOT[5] and refined with BUSTER[6] in iterative cycles. Ligand restraints were generated with GRADE[7] and MOGUL[8]. The quality of the structure was assessed with MOLPROBITY[9]. The data collection and refinement statistics are presented in Table S3.

2. Supplementary experimental: biological assay conditions

Cell lines were supplied by the German Collection of Microorganisms and Cell Cultures (DSMZ). Cell lines were authenticated by STR profiling using a GenePrint® 10 kit (Promega, Southampton, UK) and a 3730xl DNA analyser (Applied Biosystems, Warrington, UK). All STR profiles were >80% match (using ATCC or DSMZ matching algorithms) with the respective reference profile. Cells were routinely screened for *Mycoplasma*, using an in-house PCR-based assay (Universal Mycoplasma Detection Kit (30-1012K, ATCC, Manassas, VA, USA).

2.1 TR-FRET assay

Assays were performed in a 384-well black Proxiplate (Perkin Elmer) containing 1 nM Trx-6xHis-BCL6 (in house-produced, human BCL6 BTB domain covering amino-acid sequence 5-129), 300 nM BCOR-AF633 peptide (RSEIISTAPSSWVVPGP-Cys-AlexaFluor 633-amide, Cambridge Research Biochemical) and 0.5 nM anti-6xHis-Terbium cryptate (CisBio Bioassays, France), in assay buffer (25 mM Hepes pH8, 100 mM NaCl, 0.05% Tween20, 0.5 mM TCEP, 0.05% bovine serum albumin). Test compounds in DMSO or DMSO alone were added to the wells using an ECHO550 acoustic dispenser (Labcyte Inc) to give the appropriate test concentration in 0.7% v/v DMSO final. After 2 hours incubation at room temperature the plate was read on an Envision plate reader (Perkin Elmer) with 337 nm laser excitation, a first emission filter APC 665 nm and a second emission filter Europium 615 nm, or alternatively on a Pherastar FSX (BMG Labtech) plate reader equipped with 337 nm laser excitation filter, a first emission filter at 620 nm and a second emission filter at 665 nm. The % inhibition at each concentration was calculated by normalising FRET ratio to the appropriate high (DMSO with all reagents) and low (DMSO without BCL6) controls. IC_{50} values were determined using GraphPad Prism 6.0 or Dotmatics (Bishops Stortford, UK) software by fitting the normalised data to a sigmoidal four-parameter logistic fit equation.

2.2 NanoBRET Assay

A cellular nano-Bioluminescence Resonance Energy Transfer (nanoBRET) assay (Promega NanoBRET Nano-Glo Detection System, catalogue number N1662) was used to detect inhibition of the BCL6-SMRT (also called NCOR2) corepressor protein-protein interaction. DNA encoding full length BCL6 and SMRT were inserted into pFC32K.NanoLuc and pFC14K.HaloTag vectors (Promega) to produce C-terminal tagged fusion proteins BCL6-nanoLuc and SMRT-HaloTag, respectively. HEK293T cells were plated ($5x10^5$) in T75 tissue culture flask and bulk transfected 48 hours later with Fugene 6 (Promega cat# E2691) reagent and 18 µg total DNA plasmids encoding BCL6-nanoLuc as donor and SMRT-HaloTag as acceptor, at a donor:acceptor DNA ratio of 1:25. At 24 hr post-transfection, HEK293T cells were collected and stored in liquid nitrogen in 90% FBS (PAN Biotech UK) and 10% DMSO. At the time of assay, compounds (100nL/well) and NanoBRET 618 ligand (10nL of 1mg/ml stock solution per well) were dispensed in a dry 384-well NUNC white assay plate (ThermoScientific NUNC cat.#10080681) using Echo550 acoustic dispensing (Labcyte Inc.). Frozen transfected HEK293T cells were thawed, centrifuged and freezing medium was replaced by phenol red-free OptiMEM+4% FBS (Life Technology). The cell density was adjusted to $3x10^5$ cells/ml and 20 µL (6000 cells) were plated in each well containing test compounds

(0.0125-50 µM) in DMSO or DMSO alone and 0.5 µg/ml NanoBRET 618 fluorescence ligand, in 0.55% v/v DMSO final concentration. Cells were incubated for 6 hr at 37 °C / 5% CO_2 then NanoBRET furimazine substrate (Promega) was added to give a final concentration of 10 µM. After a short centrifugation the plates were read on an Envision (Perkin Elmer) plate reader equipped with a LUM/D600 Dual mirror, Lum 450/40 nm bandpass and D605 nm longpass filters, with a 0.2 sec reading to determine the BRET ratio. Alternatively, plates were read on Pherastar FSX (BMG Labtech) equipped with BRET module LP610 nm (1st emission filter) / 450-80 nm (2nd emission filter). The % inhibition at each test concentration was calculated by normalising the BRET ratio to the appropriate high and low controls. The compound IC_{50}s were determined using Graphpad Prism 6.0 or Dotmatics software by fitting the normalised data to a sigmoidal four-parameter logistic fit equation.

2.3 Cell proliferation assay

Cells were seeded in 96-well culture plates at a density of 2500 cells/well in RPMI-1640 medium (Sigma-Aldrich) supplemented with 10% FBS (Gibco). Compounds were initially dispensed into 96-well U-bottom plates using an Echo 550 acoustic dispenser (Labcyte Inc.), then diluted in RPMI-1640 medium and transferred onto the cells. Cells were treated with 8 compound concentrations in duplicate, ranging from 1.07 nM to 10 µM, in a final DMSO concentration of 0.1% and final volume of 100 µl. Cells were incubated with compound for 14 days, with medium changes at days 3, 7 and 10 carried out as follows: fresh 96-well cell culture plates were prepared containing 100 µl medium plus compound at the assay concentrations (white plates were used on day 10 to optimise luminescence measurement). Assay plates containing cells were vortexed to mix and cell density in one control well was counted using a Coulter Z2 cell counter (Beckman Coulter). The volume of medium containing 2500 cells in the control well was calculated and this volume of cells was transferred from every well of the assay plates to the corresponding well of the fresh plates containing compound. After 14 days, CellTiter Glo reagent (Promega) was added to the medium in each well of the assay plate at a ratio of 1:2, mixed on a plate shaker, then incubated at room temperature for 10 minutes. Luminescence was measured using an Envision plate reader (Perkin Elmer) and the relative luminescence at each compound concentration, compared to DMSO alone, was calculated. GI50 were determined using a 4-parameter curve fit in Dotmatics (Bishops Stortford, UK).

3. Supplementary experimental: physicochemical assays

3.1 NMR solubility assay

9 µL of 10 mM DMSO stock solution was pipetted into one well of a 384 deepwell plate (Greiner, part-no. 781270), then 171 µL of HEPES buffer (20 mM HEPES [Sigma Aldrich, cat-no. H3375-250G], 150 mM NaCl, 0.5 mM TCEP, 10% D_2O) was pipetted into the same well and mixed by up-down pipetting 3 times to create a 0.5 mM solution or suspension containing 5% DMSO. It was then separated by centrifugation (1000 rpm for 1 min, Eppendorf 5810C). The plate was then sealed and incubated at room temperature for 20 hours, without shaking. The plate was centrifuged again for 1 minute at 1000 rpm on Eppendorf 5810R before 165 µL of the supernatant was transferred to a 3 mm NMR tube (Bruker, Part No. Z112272) using liquid handler SamplePro Tube SJ S (Bruker). The concentration of the solubilized compound in solubility sample is measured by quantitative ^{1}H-NMR using a single external standard (200 µM caffeine (Sigma, C1778) in PBS (pH 7.4) with 1% DMSO-d6).

The detail of the NMR method is as following: NMR data was collected on a Bruker Avance Neo 600 spectrometer equipped with a 5 mm TCI-CryoProbe. The ^{1}H spectrum was referenced to the internal deuterated solvent. The operating frequency for ^{1}H was 600 MHz. All NMR data were acquired at the temperature of 298 K. All data were acquired and processed using Bruker Topspin 4.0. The quantitative ^{1}H-NMR spectrum was acquired using a Bruker standard 1D lc1pngppsf2 pulse sequence with 32 scans. The sweep width was 6.2 ppm with O1P set to 8.8 ppm, and the FID contained 16k time-domain data points. Relaxation delay was set to 20 sec. Water signal was suppressed. [10]

3.2 logD$_{7.4}$ assay

Calibration, validation and in-house compounds were prepared at 1mM in 10% DMSO / 90% Trizma solution (100mM Trizma in 75/25 methanol/water). 3 µL standard injections (with needle wash) of all calibration, validation and in-house samples were made onto a Phenomenex Luna C8 column (3µm, 100 x 4.6 mm, 100A, Phenomenex, Torrence, USA). Chromatographic separation at 30°C was carried out using a 1260 Series HPLC (Agilent, Santa Clara, USA) over a 5 minute gradient elution from 95:5 to 0:100 aqueous (20mM Trizma in octanol-saturated water) and organic (acetonitrile + 0.25% v/v octanol) at a flow rate of 2 mL/min. The gradient was held at 0:100 water:organic for 0.8 minutes, then returned to the starting conditions of 95:5 water:organic for 0.2 minutes. The column was re-equilibrated for 5 minutes at the

starting conditions prior to the next injection. UV-Vis spectra were acquired at 254 nm, 280 nm and 220 nm on a 1260 Series diode array detector (Agilent, Santa Clara, USA). Raw data was processed using Agilent Chemstation Rev.C.01.04.

4. Supplementary experimental: in vitro DMPK assays

4.1 Microsomal clearance assay

Microsomal clearance was determined in female CD1 mice, female Sprague–Dawley rats, and mixed gender human liver microsomes obtained from BioIVT (Peterborough, U.K.) following incubation of 1 µM compound at 37 °C in 0.5 mg/mL microsomal protein, 3 mmol/L $MgCl_2$, 1 mmol/L NADPH, 2.5 mmol/L and 10 mmol/L phosphate buffer (pH 7.4) (all purchased from Sigma-Aldrich, Gillingham, U.K). Reactions were started by addition of the cofactors following 10 min preincubation of microsomes with test compound and were terminated at −1, 0, 5, 10, 15, and 30 min with three volumes of ice-cold methanol containing internal standard. Samples were centrifuged at 2800*g* for 30 min at 4 °C and the supernatants analyzed. Control incubations were prepared as above with omission of cofactors. Compound measurements were performed by LCMS on an Agilent quadrupole time-of-flight instrument (Agilent 6510) following separation with a 3 min gradient of 0.1% formic acid in methanol on a Kinetex C18, 50 × 2.1 mm 2.6 µm UPLC column (Phenomenex, Macclesfield, UK).

4.2 Caco-2 assay

Caco-2 apparent permeability (Papp) was determined in the Caco-2 human colon carcinoma cell line. Cells were maintained in DMEM with 10% fetal bovine serum, penicillin, and streptomycin in a humidified atmosphere with 5% CO_2/95% air for 10 days. Cells were then plated onto a cell culture assembly plate (Millipore, UK), and monolayer confluency was tested using a TEER electrode prior to the assay. Media was washed off and replaced with HBSS buffer (pH7.4) containing compound (1 (or 10 µM where specified), 1% DMSO) in the appropriate apical and basal donor wells. HBSS buffer alone was placed in acceptor wells. The Caco-2 plate was incubated for 2 h at 37 °C. Samples from the apical and basolateral chambers were analyzed using a Waters (Milford, MA, US) TQ-S LC-MS/MS system.

Apparent permeability (Papp) was determined as follows:

$$\mathbf{P_{app}} = \frac{\mathbf{V_r\ (mL)}}{\mathbf{A\ (cm^2) \times C_0\ (\mu M)}} \times \mathbf{Rate\ of\ Diffusion\ (\mu M.sec^{-1})}$$

Where V_r = volume of receptical

A = surface area of monolayer

C_0 = Initial compound concentration in donor

4.3 Protein binding assay

Protein binding in mouse plasma (Charles River, Wilmington, MA, USA) and in Iscove's Modified Dulbecco's Medium (IMDM, Gibco, Life technologies Ltd, Paisley, UK) + 10% FBS was measured using Rapid Equilibrium Dialysis (RED, Thermo Fisher Scientific, Loughborough, UK). Test compound in DMSO was spiked into either mouse plasma or cell culture media containing protein as appropriate resulting in a concentration of 5 µM for dialysis, containing 1 % DMSO. 300 µL of spiked matrix was added to the donor side of the RED plate and 500 µL of dialysate (either 100 mM phosphate buffer or protein free media for plasma or media protein binding respectively) was added to the receiver well. Dialysis was performed by shaking for 4 h at 37 °C. After dialysis, samples were matrix matched followed by protein precipitation with acetonitrile containing internal standard. Samples were mixed, centrifuged and supernatant was taken for analysis by ESI-LCMS/MS on a Waters (Milford, MA, USA) Xevo TQ-S following gadient separation with 0.1% formic acid in water and acetonitrile on a Phenomenex (Macclesfield, UK) Kinetex C18 UPLC column (50 × 2.1 mm, 2.6 µM).

The fraction unbound (f_u) was calculated as follows:

$f_u' = \frac{PAR\ receiver}{PAR\ donor}$ where PAR = Peak Area Ratio of Analyte/Internal Standard.

$$f_u = 1/(1 + \left(\frac{1}{fu'} - 1\right))$$

5. <u>Supplementary experimental: in vivo PK and PD experiments</u>

All procedures were in accordance with UK Home Office regulations under the Animals (Scientific Procedures) Act 1986, approved by The Institute of Cancer Research's Ethics Committee and in accordance with published guidelines.[11]

5.1 *In vivo* pharmacokinetic studies

Animals were adapted to laboratory conditions for at least 1 week prior to dosing and were allowed food and water *ad libitum*. Compounds were administered iv or po (mouse, 0.1 mL/10 g in 10% DMSO, 5% tween 20 in saline); blood samples were collected in heparinised capillaries from the tail vein at 7 or 8 time points over 6 or 24 h post dose and frozen on collection together with a standard curve and quality controls spiked in control blood. Samples were reconstituted in a water:MeOH mixture containing internal standard as previously described (Roberts et al, 2016). Following centrifugation, extracts were analyzed by multiple reaction monitoring of precursor and product ions by ESI-LCMS/MS on either a Waters (Milford, MA, USA) Xevo TQ-S or Sciex (Framingham, MA, USA) QTrap6500 following gradient separation with 0.1% formic acid and methanol on a Phenomenex (Macclesfield, UK) Kinetex C18 UPLC column (50 × 2.1 mm, 2.6 μM). Quantitation was carried out with an external calibration. Quality controls were included and were within 20% of nominal concentration.

Pharmacokinetic parameters were derived from noncompartmental analysis using Phoenix Pharsight Non compartmental analysis (model 200 and 201) version 6.3. All parameters are calculated from timepoints up to 6 h.

5.2 Formulation of CCT374705 (17)

A solution formulation suitable for higher concentrations (> 5 mg/mL) of **CCT374705** was developed by SEDA as described in Table S4.

CCT374705 was dissolved in a pre-determined volume of DMSO (Sigma-Aldrich, UK) and to this was added a pre-determined volume of Kolliphor HS15 (Sigma-Aldrich, UK, 42966) at 40 °C. The solution was briefly vortexed before addition of a pre-determined volume of PEG400 (Sigma-Aldrich, UK, 8.07485) at 40 °C. After a brief vortex a pre-determined volume of aqueous HPMC (1.25%, viscosity 40-60cp grade, Sigma-Aldrich, UK, H8384) was added. The formulation was vortexed and then sonicated at 40 ^{o}C for ~45-60 mins. Complete vehicle was prepared in the same manner with no compound added. The formulation was stored at room temperature for 3-4 days maximum. Both compound and vehicle solutions were delivered orally (0.2 mL per 20 g mouse) using a gavage needle - mice were individually weighed and dosing volumes adjusted accordingly.

5.3 *In vivo* pharmacodynamic (PK/PD) and efficacy studies

5.3.1 Preparation of Karpas 422 Cells

The Karpas 422 cells (B-cell lymphoma cells) cell line was supplied by Public Health England, UK (ECACC) and authenticated by standard tandem repeat (STR) profiling and screened for mycoplasma using an in-house PCR-based assay. The cells were grown in RPMI-1640 medium (Gibco UK), supplemented with 10% FBS (Sera Plus, PAN Biotech UK) for ~12 days post resuscitation from a viable freeze. Cells were maintained in a humidified incubator, set at 37 °C and 5% CO2. Cell suspensions were replenished with fresh growth medium every 2-3 days and grown to density of 0.7-1 million cells per mL before harvesting for an *in vivo* study. All cultured cells were centrifuged (~150g) and the pellet resuspended in cold serum-free RPMI-1640 [supplemented with bFGF, Sigma #F0291 (final 150ng/mL) & VEGF, Sigma #V7259 (final 50ng/mL)] to a concentration of 10^8/mL. An equal volume of cold Matrigel (Corning #354324) was added, final concentration is at $5x10^7$/mL. Cells were maintained on wet ice prior to subcutaneous injection at 10^7/200uL, bilaterally, to female SCID mice (~5-6 weeks old, Charles River, UK).

5.3.2 Establishing a Karpas 422 subline

Fastest growing tumours were excised to a sterile petri dish and minced crossways with two scalpel blades. The minced tissue was lifted to a bijou containing 0.5mL of Liberase TM (final @ 50ug/mL) (Roche, #05401119001) plus 4.5mL HBSS (Sigma #H9394). The mix was digested for ~1 hour in a water bath set at 37 °C and briefly vortexed every twenty minutes, before washing twice and finally resuspending in growth medium supplemented with Primocin (#ant-pm-05, anti-microbe, InvivoGen, UK) (final @ 100ug/mL). Cells were incubated for three days before removing the Primocin; further expansion continued in normal growth medium. This subline was labelled as Karpas 422A and returned to SCID mice as described above. Again, the fastest growing tumours were selected and digested as described above. This subline was labelled as Karpas 422B and returned to SCID mice from which 100% tumour take-rates were yielded with survival at approximately 60-70 days.

5.3.3 Preparation of Karpas 422 xenograft model in mouse

Karpas 422B cells were prepared for injection at a final concentration of 5 x10^7 cells/mL, using serum–free RMPI-1640 and an equal volume of Matrigel®, (#354234, Corning® B.V., Netherlands) both chilled to 4°C. Cells were delivered to female SCID mice (NOD.CB17-*Prkdc^{scid}*/J, Charles River, UK) at 10^7 per 200uL, subcutaneously, single site. At three weeks post injection tumour bearing mice (mean diameter 0.5 cm^2 +/- 0.1) were randomly selected and assigned to a treated group and a control group (n = 10 per group).

CCT374705 and vehicle were formulated as described in **Supplementary experimental 5.2: CCT374705** (50 mg/kg) and vehicle (0.2 mL/20g body weight) were administered orally, twice per day, approximately 12 hours apart for 35 days.

5.3.4 Tissue sampling

Blood (~1 mL) was taken from anaesthetized mice, via intracardiac puncture, using a 27 gauge needle and a syringe pre-coated with heparin (200 Units/mL), The heparinized blood was microcentrifuged for 2 minutes and ~200 uL of plasma was aspirated to a labelled tube, frozen on dry-ice and dedicated to pharmacokinetic analysis (PK). Each tumour was excised, divided into two halves, weighed and then snap

frozen in liquid nitrogen, dedicating one half to PK analysis and the other half to pharmacodynamic analysis (PD). All frozen material was permanently stored in a -80°C freezer.

5.3.5 *Tumour lysis and Wes method*

Tumours were lysed in lysis buffer (50 mM Tris-HCl, pH 7.4, 150 mM NaCl, 1% Triton X-100 (v/v), 1 mM PMSF, 1 mM activated sodium vanandate, 1 mM EDTA, phosphatase inhibitors 2 and 3 (1:50m dilution) and protease cocktail (1:100 dilution)) using reinforced homogenizing tubes containing metal beads using a Precellys 24 with cryolys cooling at 6000 rpm, 2 × 20 s (Stretton Scientific). Lysates were incubated on ice for 15 minutes, spun at 14000 rpm at 4 °C for 10 minutes and supernatants collected and aliquoted. Samples were stored at -80 ^{o}C until use. Protein concentration was determined by diluting 1:10 with lysis buffer and measuring on Direct Detect® spectrometer according to manufacturer's instructions.

Protein lysates were diluted to 0.0875 mg/ml with 0.1x sample buffer and run on Wes 12-230 kDa kit according to manufacturer's instructions (ProteinSimple) with multiplexing BCL6 (CST14895 at 1:150 dilution) and GAPDH (CST 2118 at 1:50 dilution). Data was analysed using Compass for SW software. BCL6 data was normalised to GAPDH and transferred to GraphPad Prism 7.

5.3.6 *Quantitative RT-PCR*

Xenograft tumour samples were lysed in Total RNA lysis solution (ThermoFisher Scientific) with mechanical disruption using a Precellys homogeniser (Bertin Instruments). RNA was purified using a MagNA Pure 96 instument according to the manufacturer's protocol (Roche). cDNA was prepared from 1 µg total RNA using qScript XLT cDNA supermix (Quantabio). ARID3A expression normalized to GAPDH was measured using a ViiA7 384-well qPCR machine (Applied Biosystems) with TaqMan Fast Advanced master mix and a TaqMan probes targeting ARID3A (Hs00193296_m1) and GAPDH (Hs02758991_g1) (ThermoFisher Scientific). ARID3A expression relative to vehicle-treated tumours was determined using the $2^{-\Delta\Delta Ct}$ method.

6. Analytical data for CCT374705

6.1 LCMS method

LC/MS and HRMS analysis for compounds shown below was performed on an Agilent 1200 series HPLC and diode array detector coupled to a 6210 time of flight mass spectrometer with dual multimode APCI/ESI source. Analytical separation was carried out at 30 °C on a Merck Chromolith Flash column (RP-18e, 25 x 2 mm) using a flow rate of 0.75 mL/min in a 4 minute gradient elution with detection at 254 nm. The mobile phase was a mixture of methanol (solvent A) and water (solvent B), both containing formic acid at 0.1%. Gradient elution was as follows: 5:95 (A/B) to 100:0 (A/B) over 2.5 min, 100:0 (A/B) for 1 min, and then reversion back to 5:95 (A/B) over 0.1 min, finally 5:95 (A/B) for 0.4 min.

6.2 HPLC and LCMS traces for CCT374705

High resolution LCMS trace of ***CCT374705***

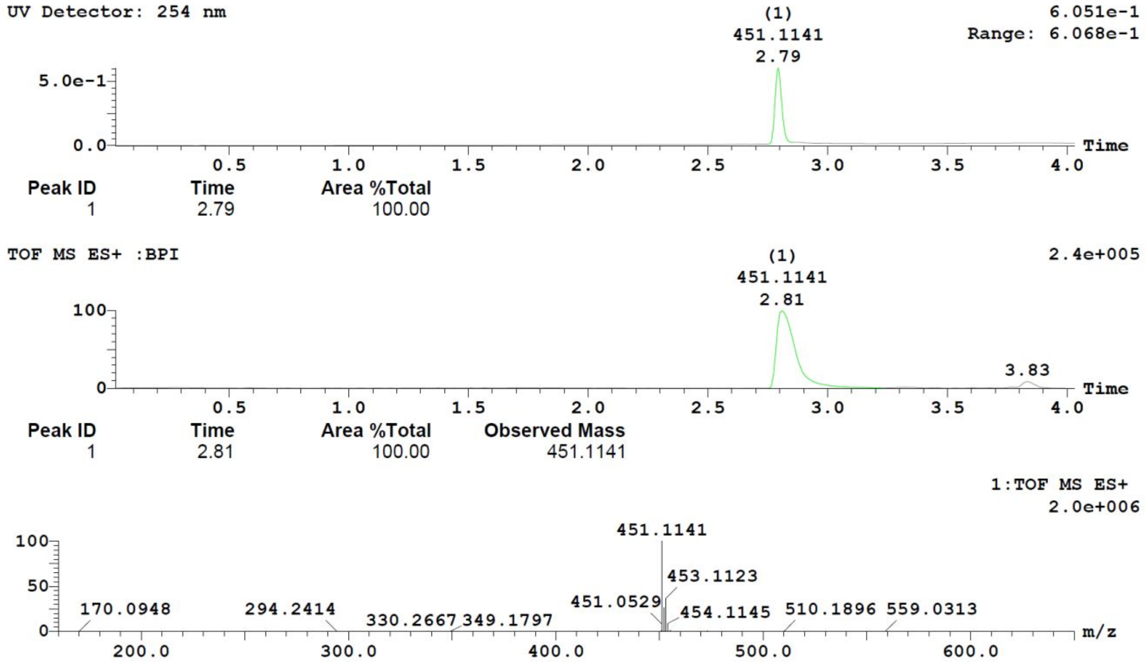

6.3 qNMR method

Sample Preparation

Around 2 mg of in-house compound or caffeine (Sigma reference standard, C1778) was accurately weighed (Mettler Toledo XP26 micro balance) and dissolved in 500 µL of deuterated DMSO (Euriso-top, D034T). 2 samples were prepared for each compound. 160 uL of the solution was transferred to 3 mm NMR tube (Bruker Z112272), 3 tubes were prepared from the same sample.

NMR experiment

NMR data was collected on a Bruker Avance NEO spectrometer equipped with 600 MHz magnet and 5 mm TCI Cryoprobe. The ^{1}H spectrum was referenced to the internal deuterated solvent. The operating frequency for ^{1}H was 600 MHz. All NMR data were acquired at the temperature of 298 K. All data were acquired and processed using Bruker Topspin 4.0. The quantitative 1H-NMR spectrum (@qHNMR3) was acquired using a Bruker standard 1D zg pulse sequence with 32 scans. The sweep width was 19.8 ppm, and the FID contained 64k time-domain data points. Relaxation delay was set to 20 sec. The average value of the integrals in the aromatic region (7-9 ppm) was used to obtain the absolute concentration of the compound. Quantitative Data was analysed using Topspin 4.0.5, Eretic function. For each sample, three NMR tubes were prepared as described above, and the averaged value was used to obtain the purity value for each sample. The average purity of the two samples was quoted as the final compound purity. The purity of caffeine control samples (98.3%) was consistent with its specification (98.5-101.0%). The SD between three NMR tubes was less than 3% and the SD between two samples was 0.2%.

6.4 qNMR analysis for CCT374705

The purity analysis of **CCT374705** was found to be 98.2% (SD 5.2%) (Table SX).

Example ^{1}H NMR spectrum used in CCT374705 qNMR analysis

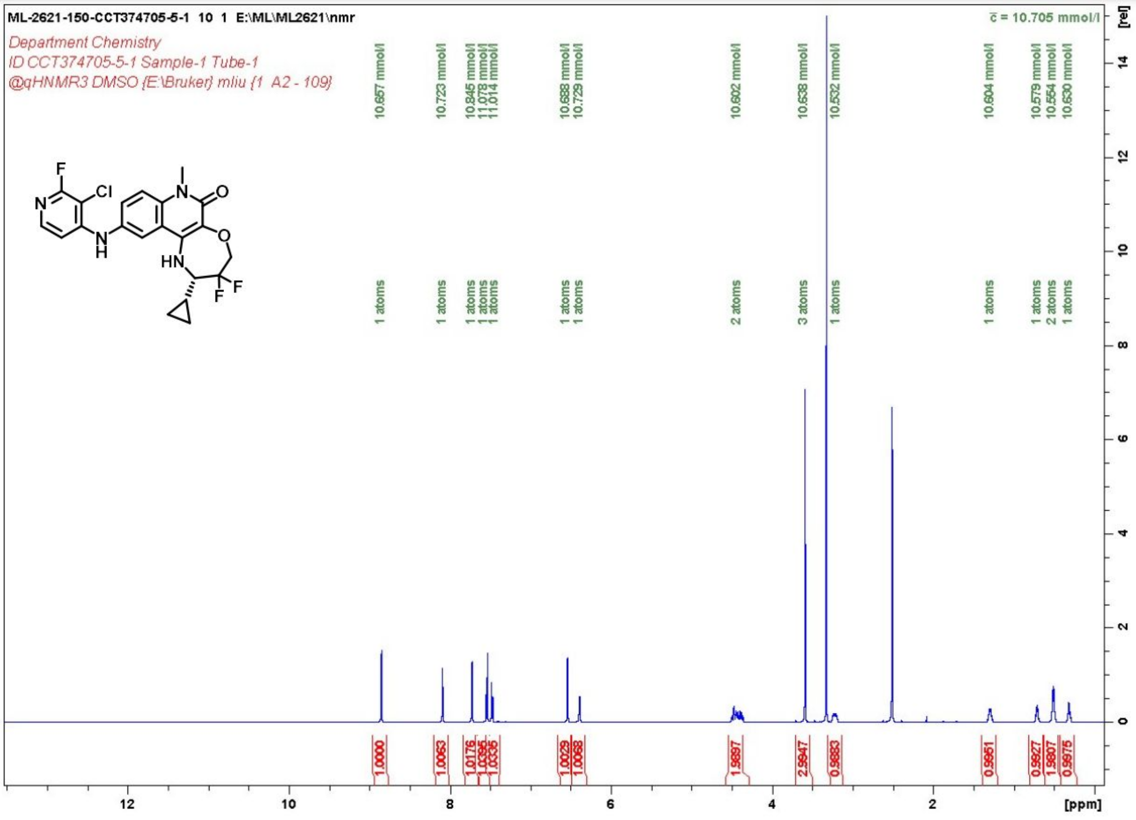

7. Supplementary Tables

Supplementary Table S1:

TR-FRET summary statistics and individual replicate values

No.	BCL6 TR-FRET IC_{50} Geometric Mean (nM)	BCL6 TR-FRET IC_{50} individual replicates (nM)	BCL6 TR-FRET pIC_{50} mean	BCL6 TR-FRET pIC_{50} SD	BCL6 TR-FRET pIC_{50} SEM	BCL6 TR-FRET IC_{90} Geometric Mean (nM)	BCL6 TR-FRET IC_{90} individual replicates (nM)	n
3	3.2	3.2, 3.5, 3.1	8.4896	0.0266	0.0154	21.1	22.1, 21.0, 20.3	3
4	1.5	1.7, 1.4, 1.4, 1.8, 1.2, 1.7	8.8212	0.0691	0.0282	10.2	12.7, 7.3, 15.7, 11.0, 6.6, 10.8	6
5	3	2.6, 2.6, 3.8	8.527	0.0959	0.0554	19.9	14.2, 21.0, 26.4	3
6	0.9	0.8, 1.2, 0.8	9.0422	0.12	0.0693	4.6	5.9, 3.6, 4.6	3
7	1.9	1.6, 2.5, 1.6	8.728	0.1153	0.0666	13.7	11.0, 16.7, 13.9	3
8	1.3	1.6, 1.3, 1.1	8.8826	0.0819	0.0473	7.6	6.3, 12.8, 5.5	3
9	5.5	5.4, 4.5, 6.9	8.2581	0.0922	0.0532	45.4	42.4, 61.4, 36.0	3
10	3.9	4.9, 3.8, 4.5, 2.9, 3.9, 3, 4.9	8.4059	0.0929	0.0310	34.1	33.7, 25.4, 24.6, 40.9, 31.2, 38.9, 21.6	9
11	10.3	9.4, 11.1, 10.4	7.9892	0.0362	0.0209	85.4	109.8, 70.4, 80.4	3
12	5	7.3, 5.2, 3.3	8.3025	0.1736	0.1002	46.6	38.2, 64.5, 41.1	3
13	6.2	7, 8.1, 7, 3.7, 6.3	8.2073	0.1341	0.0600	50.2	63.3, 62.0, 34.3, 53.5, 44.4	5
CCT372064, 14	3.9	2.4, 16.2, 2.9, 2.9, 2.9, 4, 3.9	8.4036	0.2813	0.1063	30.1	17.6, 34.1, 29.8, 28.5, 17.0, 118.9, 21.6	7
15	13.4	12.4, 13.9, 13.9	7.873	0.0292	0.0169	108.6	13.9, 13.9, 12.4	3
16	18.5	16.9, 20.2	7.7332	0.0548	0.038	158.3	177.4, 141.4	2
CCT374705, 17	6.2	14.2, 5.3, 3.9, 8.9, 5.2, 4.2, 6, 6.8, 4.9, 4.7, 4.5, 7.3, 9.9, 5.9, 6, 7.3	8.2095	0.1492	0.0373	56.7	32.4, 63.9, 57.4, 78.6, 40.5, 41.3, 45.7, 145.0, 109.2, 47.4, 66.4, 90.4, 47.2, 38.9, 40.8, 46.7	16
18	20.7	23.9, 16.5, 22.6	7.6837	0.0871	0.0503	201.4	233.8, 211.6, 165.1	3

Supplementary Table S2:

nanoBRET summary statistics and individual replicate values

No.	BCL6 NanoBRET IC_{50} Geometric Mean (nM)	BCL6 NanoBRET IC_{50} individual replicates (nM)	BCL6 NanoBRET pIC_{50} mean	BCL6 NanoBRET pIC_{50} SD	BCL6 NanoBRET pIC_{50} SEM	BCL6 NanoBRET IC_{90} individual replicates (nM)	BCL6 NanoBRET IC_{90} Geometric Mean (nM)	n
3	15.3	11.9, 14, 21.5	7.8146	0.1319	0.0762	121.4	50.5, 110.5, 320.2	3
4	10.7	12.3, 8.2, 12.1	7.9696	0.0991	0.0572	142.5	185.1, 101.2, 154.4	3
5	12.5	9.6, 14.7, 14	7.9026	0.1007	0.0581	98.7	47.4, 140.1, 144.8	3
6	26.9	27, 30, 24.1	7.57	0.0481	0.0278	218.0	188.9, 229.1, 239.4	3
7	41	28.2, 47.9, 51.1	7.3871	0.1417	0.0818	284.2	200.2, 212.8, 538.5	3
8	4.5	3.5, 4, 6.5	8.3472	0.1394	0.0805	25.0	25.0, 15.4, 40.8	3
9	149.3	229.4, 48.1, 301.8	6.8258	0.4302	0.2484	1621.0	2188.8, 474.0, 4106.1	3
10	43.6	26, 57.5, 55.3	7.3608	0.1944	0.1122	386.1	175.5, 326.2, 1005.7	3
11	31	61.1, 18.2, 26.8	7.5086	0.2688	0.1552	174.8	411.0, 111.1, 117.0	3
12	54.7	73.6, 63.9, 34.7	7.2623	0.1735	0.1002	385.1	531.9, 397.8, 269.9	3
13	52	56.7, 28.4, 87.3	7.284	0.2461	0.1421	443.6	642.7, 155.8, 872.0	3
CCT372064, 14	11.7	14.7, 10.4, 10.4	7.9327	0.0858	0.0495	124.3	222.8, 107.8, 79.9	3
15	68.9	105.9, 43.5, 71	7.1618	0.1935	0.1117	796.5	729.2, 571.0, 1213.8	3
16	128.6	128.6	6.8907	-	-	639.4	639.4	1
CCT374705, 17	21.7	15.4, 10.1, 12.1, 44.2, 19.7, 14.6, 22.2, 25.2, 25.1, 17.3, 24.3, 18.4, 20.9, 21.8, 24.2, 77.1, 15.6, 11.1, 40.7, 25.3, 23.2, 13, 32.6, 29.5	7.6643	0.2022	0.0413	140.5	90.8, 61.5, 38.7, 509.5, 106.0, 75.2, 246.4, 226.8, 142.9, 106.7, 149.6, 241.2, 109.2, 136.1, 155.2, 583.1, 149.0, 47.8, 257.1, 240.9, 137.8, 65.2, 115.0, 212.4	24
18	107.5	99.9, 125.1, 99.5	6.9685	0.0569	0.0329	616.0	625.4, 510.8, 731.6	3

Supplementary Table S3:

Crystallographic data collection and refinement statistics.

Crystal system	Flag-BCL6
Ligand	**CCT374705**
PDB code	8C78
Crystal	
Space group	P 6_1 2 2
Unit cell dimensions (a/b/c in Å)	67.59/67.59/167.16
Unit cell angles (α/β/γ in °)	90/90/120
Data collection and processing	
Beamline	DLS I03
Wavelength (Å)	0.9763
Integration program	DIALS
Reduction program	AIMLESS
Resolution range	47.95 – 1.80
Number of unique reflections [a]	21872 (1262)
Completeness [a]	100 (99.7)
Redundancy [a]	25.4 (28.1)
R_{merge} (%) [a]	7.3 (255.0)
I/σ(I) [a]	24.3 (1.2)
$CC_{1/2}$ [a, b]	0.988 (0.725)
Refinement	
Program	BUSTER
R_{work} (%)	19.88
R_{free} (%)	23.21
Number of residues	130
Number of water molecules	150
Average B-factor ($Å^2$)	47.73
Ramachandran favoured (%)	97.66
Ramachandran outliers (%)	0
RMSD bonds (Å)	0.011
RMSD angles (°)	1.407

[a] Values in parentheses are for the highest resolution shell.

[b] Half-dataset correlation coefficient, see: Karplus, P. A.; Diederichs, K. Linking crystallographic model and data quality. *Science* **2012**, *336*, 1030−1033.

Supplementary Table S4:

Composition of solution formulation for ***CCT374705***

Component	Amount (%)	Amount per 10 mL (5 mg/mL)
**CCT374705**		50 mg
**DMSO**	10% v/v	1 mL
**Kolliphor HS15**	20% v/v	2 mL
**PEG400**	30% v/v	3 mL
**HPMC 1.25% aq**	40% v/v	4 mL

Supplementary Table S5:

qNMR purity analysis for ***CCT374705***

Compound	Batch	Sample	tube	Weight (mg)	MW	Volume (uL)	Calc Conc. (mM)	qNMR Conc. (mM)	Purity (%)	Average 3 tubes	SD 3 tubes	Average 2 samples	SD 2 samples
CCT374705	5	1	1	2.266	450.85	500	10.052	10.501	104.5%	101.9%	2.3%	98.2%	5.2%
CCT374705	5	1	2	2.266	450.85	500	10.052	10.191	101.4%				
CCT374705	5	1	3	2.266	450.85	500	10.052	10.047	99.9%				
CCT374705	5	2	1	2.246	450.85	500	9.963	9.445	94.8%	94.5%	3.9%		
CCT374705	5	2	2	2.246	450.85	500	9.963	9.796	98.3%				
CCT374705	5	2	3	2.246	450.85	500	9.963	9.015	90.5%				

8. Supplementary Figures

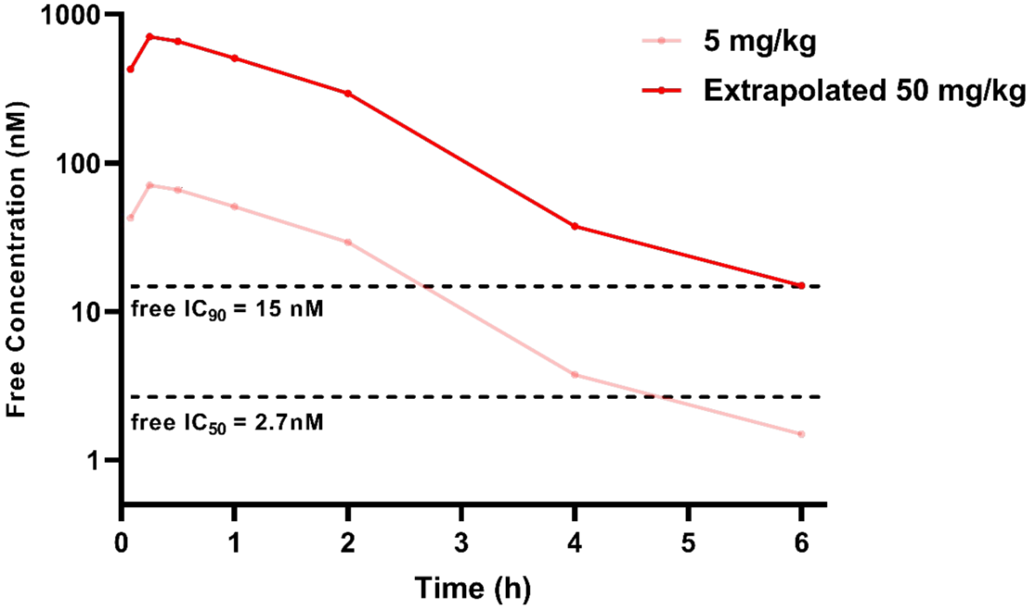

Supplementary Figure S1:

*Free mean mouse (BALB/c) blood concentrations (nM) of **8** after po dosing at 5 mg/kg and extrapolated (assuming linear PK) to 50 mg/kg PO dosing. The dashed, black lines represent the calculated free, cellular (from nanoBRET assay) IC_{50} and IC_{90} values (nM).*

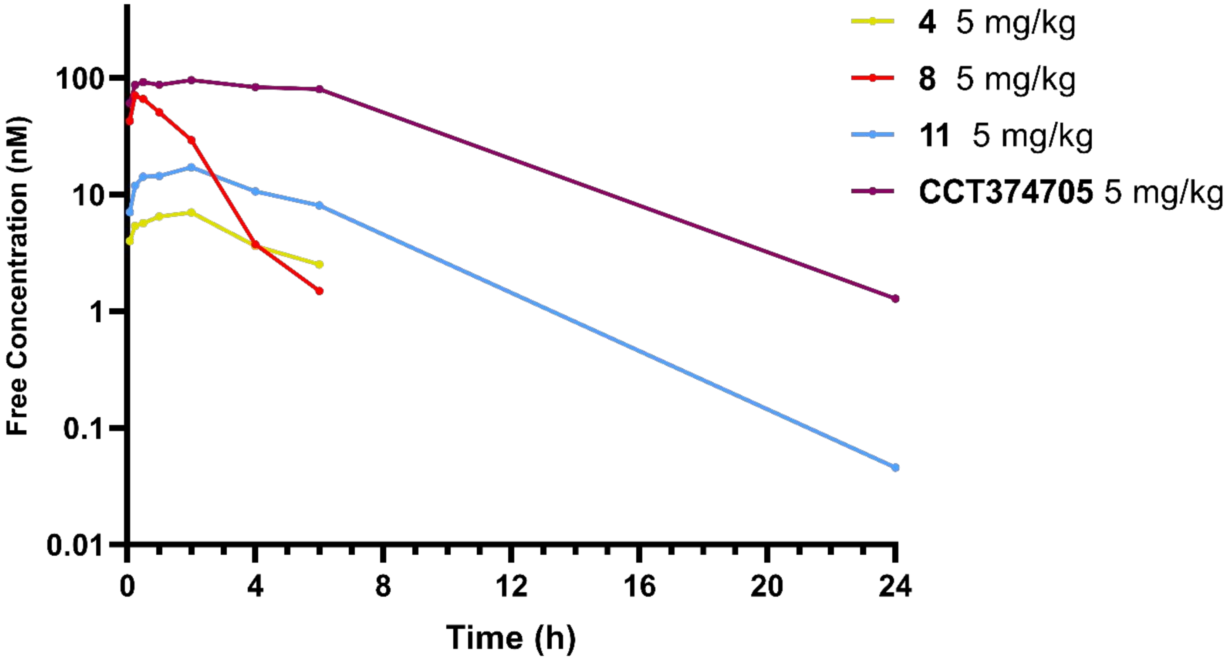

Supplementary Figure S2:

*Free mean mouse (BALB/c) blood concentrations (nM) of **8** (red), **4** (yellow), **11** (blue) and **CCT374705 (17)** (purple) after po dosing at 5 mg/kg.*

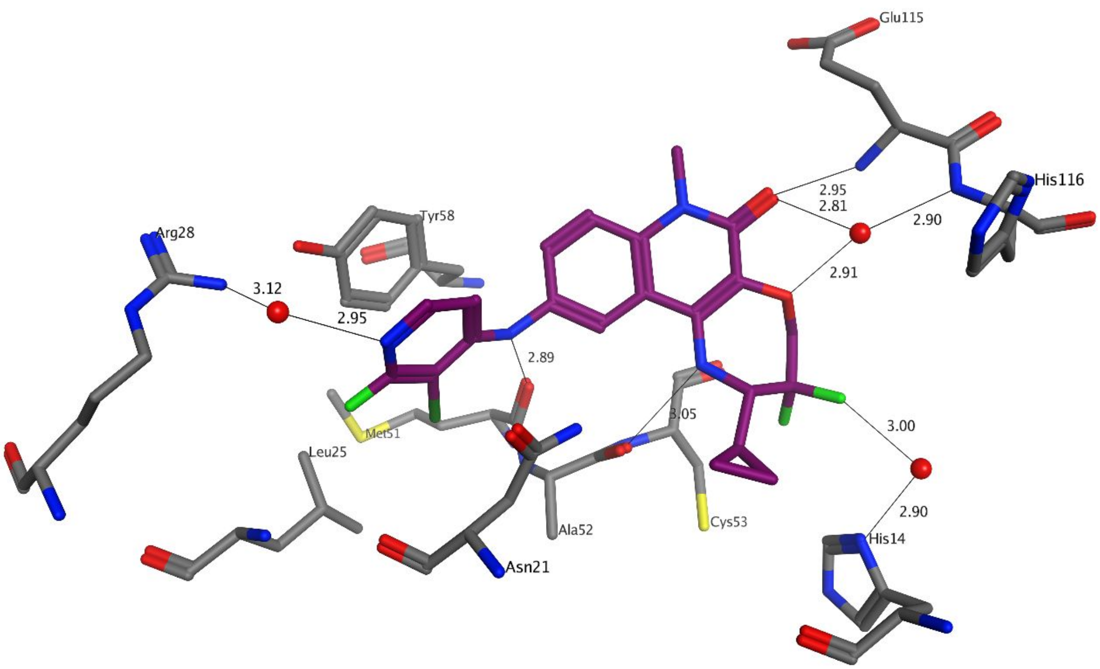

Supplementary Figure S3:

*X-ray structure of the BCL6 BTB domain with bound ligand **CCT374705** (PDB: 8C78, purple) highlighting key distances between protein and ligand.*

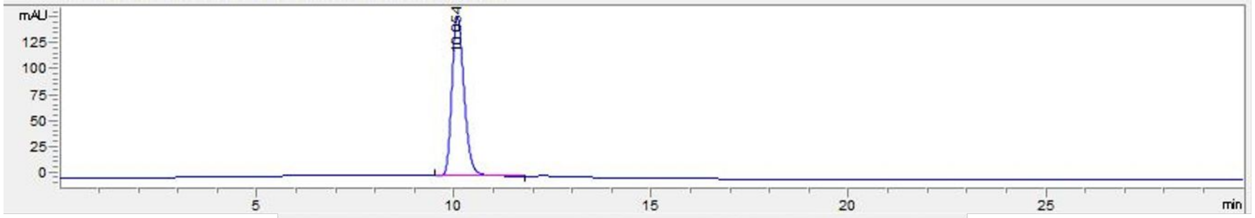

#	Time	Area	Height	Width	Area%	Symmetry
1	10.054	3331.7	154.6	0.3334	100.000	0.727

**Column Details**	Lux Cellulose-4 column (4.6mm x 250mm, 5µm)
**Flow Rate**	1 ml/min
**Detector Wavelength**	190-400 nm
**Injection Volume**	2.0 µL
**Conditions**	100% MeCN 30 min

Supplementary Figure S4:

*Chiral HPLC analysis of **CCT374705***

9. References for supplementary information

(1) Winter, G.; Waterman, D. G.; Parkhurst, J. M.; Brewster, A. S.; Gildea, R. J.; Gerstel, M.; Fuentes-Montero, L.; Vollmar, M.; Michels-Clark, T.; Young, I. D.; et al. <i>DIALS</i>: implementation and evaluation of a new integration package. *Acta Crystallographica Section D Structural Biology* **2018**, *74* (2), 85-97. DOI: 10.1107/s2059798317017235.
(2) Evans, P. Scaling and assessment of data quality. *Acta Crystallographica Section D* **2006**, *62* (1), 72-82. DOI: doi:10.1107/S0907444905036693.
(3) McCoy, A. J.; Grosse-Kunstleve, R. W.; Adams, P. D.; Winn, M. D.; Storoni, L. C.; Read, R. J. Phaser crystallographic software. *Journal of Applied Crystallography* **2007**, *40* (4), 658-674. DOI: doi:10.1107/S0021889807021206. Winn, M. D.; Ballard, C. C.; Cowtan, K. D.; Dodson, E. J.; Emsley, P.; Evans, P. R.; Keegan, R. M.; Krissinel, E. B.; Leslie, A. G. W.; McCoy, A.; et al. Overview of the CCP4 suite and current developments. *Acta Crystallographica Section D* **2011**, *67* (4), 235-242. DOI: doi:10.1107/S0907444910045749.
(4) Ghetu, A. F.; Corcoran, C. M.; Cerchietti, L.; Bardwell, V. J.; Melnick, A.; Privé, G. G. Structure of a BCOR corepressor peptide in complex with the BCL6 BTB domain dimer. *Molecular Cell* **2008**, *29* (3), 384-391. DOI: 10.1016/j.molcel.2007.12.026.
(5) Emsley, P.; Cowtan, K. Coot: model-building tools for molecular graphics. *Acta Crystallographica Section D* **2004**, *60* (12 Part 1), 2126-2132. DOI: doi:10.1107/S0907444904019158.
(6) G. Bricogne, E. B., M. Brandl, C. Flensburg, P. Keller, W. Paciorek, P. Roversi,; A. Sharff, O. S. S., C. Vonrhein, T.O. Womack. *BUSTER, Version 2.10.2, Global Phasing Ltd., Cambridge, United Kingdom* **2015**.
(7) O.S. Smart, T. O. W., A. Sharff, C. Flensburg, P. Keller, W. Paciorek,; C. Vonrhein, G. B. *Grade, Version 1.2.9, Global Phasing Ltd., Cambridge, United Kingdom, 2014.*
(8) Bruno, I. J.; Cole, J. C.; Lommerse, J. P. M.; Rowland, R. S.; Taylor, R.; Verdonk, M. L. IsoStar: A library of information about nonbonded interactions. *Journal of Computer-Aided Molecular Design* **1997**, *11* (6), 525-537, journal article. DOI: 10.1023/a:1007934413448.
(9) Chen, V. B.; Arendall, W. B., III; Headd, J. J.; Keedy, D. A.; Immormino, R. M.; Kapral, G. J.; Murray, L. W.; Richardson, J. S.; Richardson, D. C. MolProbity: all-atom structure validation for macromolecular crystallography. *Acta Crystallographica Section D* **2010**, *66* (1), 12-21. DOI: doi:10.1107/S0907444909042073. Davis, I. W.; Leaver-Fay, A.; Chen, V. B.; Block, J. N.; Kapral, G. J.; Wang, X.; Murray, L. W.; Arendall, W. B., III; Snoeyink, J.; Richardson, J. S.; et al. MolProbity: all-atom contacts and structure validation for proteins and nucleic acids. *Nucleic Acids Research* **2007**, *35* (suppl_2), W375-W383. DOI: 10.1093/nar/gkm216 (acccessed 10/4/2019).
(10) Wider, G.; Dreier, L. Measuring Protein Concentrations by NMR Spectroscopy. *Journal of the American Chemical Society* **2006**, *128* (8), 2571-2576. DOI: 10.1021/ja055336t. Lin, M.; Tesconi, M.; Tischler, M. Use of 1H NMR to facilitate solubility measurement for drug discovery compounds. *International Journal of Pharmaceutics* **2009**, *369* (1), 47-52. DOI: https://doi.org/10.1016/j.ijpharm.2008.10.038. Saal, C.; Petereit, A. C. Optimizing solubility: Kinetic versus thermodynamic solubility temptations and risks. *European Journal of Pharmaceutical Sciences* **2012**, *47* (3), 589-595. DOI: http://dx.doi.org/10.1016/j.ejps.2012.07.019.

(11) Workman, P.; Aboagye, E. O.; Balkwill, F.; Balmain, A.; Bruder, G.; Chaplin, D. J.; Double, J. A.; Everitt, J.; Farningham, D. A. H.; Glennie, M. J.; et al. Guidelines for the welfare and use of animals in cancer research. *British Journal of Cancer* **2010**, *102* (11), 1555-1577. DOI: 10.1038/sj.bjc.6605642.
